# Impact of Veterinary Herd Health Management on German Dairy Farms: Effect of Participation on Farm Performance

**DOI:** 10.3389/fvets.2022.841405

**Published:** 2022-04-07

**Authors:** Jenny Ries, Katharina Charlotte Jensen, Kerstin Elisabeth Müller, Christa Thöne-Reineke, Roswitha Merle

**Affiliations:** ^1^Institute for Veterinary Epidemiology and Biostatistics, Department of Veterinary Medicine, Freie Universität Berlin, Berlin, Germany; ^2^Ruminant and Swine Clinic, Department of Veterinary Medicine, Freie Universität Berlin, Berlin, Germany; ^3^Institute of Animal Welfare, Animal Behavior and Laboratory Animal Science, Department of Veterinary Medicine, Freie Universität Berlin, Berlin, Germany

**Keywords:** survey, dairy herd health management, cooperation with veterinarian, integrated herd health management, future of dairy farming

## Abstract

German dairy farming has intensified markedly in recent years, and the demand for Veterinary Herd Health Management (VHHM) is rising. To protect farms from epidemics, ensure food safety, and prevent developing of antibiotic resistance, VHHM has been anchored in EU law since April 2021. Via an online survey, distributed by different farmers' organizations, dairy farmers were asked about the cooperation with their veterinarian. The aim was to evaluate farm performance as a function of participation in VHHM. From 216 analyzed questionnaires, 106 respondents participated in VHHM. Results showed that farmers who make use of VHHM and consult their veterinarian in decision-making frequently have the highest 305-day milk yield (305dMY), the lowest bulk tank somatic cell counts, and the lowest age at first calving (AFC). However, these farmers tended to have higher replacement rates and a higher mortality of cows in the period up to 60 days in milk (MORT60DIM). Furthermore, respondents who defined VHHM as “evaluation of herd data, strategic planning” had the highest 305dMY compared with those who defined VHHM through one of the different options given (“pregnancy checks and support in reproduction”/“problem solving”). In the multifactorial regression model, VHHM participating farms had a 660-kg higher 305dMY and 1 month less in AFC, compared with farms not participating in VHHM. However, within the VHHM participants, no association between VHHM practices and performance parameters was found. Further research is needed, to find out if tailored advice of the VHHM approach may show effect herein.

## Introduction

Dairy farming is of utmost economic importance in Germany. In 2020, Germany was the largest EU milk producer with an output of approximately 33 million tons of milk. Approximately half of the produced quantity was exported, representing a production value of more than 10 billion € (1). The increase in quantities is inherent in structural changes on dairy farms demanding intensification with, as a result, an uneven decline in the number of farms and animals (2–4). Since the discontinuation of the milk quota in 2015, the dependency has made the milk price a pawn on the global market (5). In parallel to the economic pressure at the beginning of the production chain, legal and social requirements also set the expectations on dairy farms even higher (6–8). For this reason, farmers must improve animal health and welfare while optimizing their milk production to continue to withstand economic constraints and survive in the marketplace.

Over the years, breeding progress has made a considerable contribution to the development of today's dairy industry. This development led to an increased milk yield but also to an increased incidence of production associated diseases, such as decreased fertility, milk fever, hyperketonemia, and so on (9–11). In addition, societal demands on dairy farming have become louder, and consumers prefer small-structured farms without exploitation of animals and the environment (12). Consequently, stakeholders of agriculture need to be aware that only transparency can counteract societal alienation from modern agriculture (13, 14).

Considering the developments mentioned above, an all-encompassing approach is needed not only to balance the extreme demands on our dairy cows with the production-associated diseases, but also to ensure animal welfare and food safety for the future of German dairy farming, not only to appease the consumer. Therefore, Veterinary Herd Health Management (VHHM) programs have been established since on-farm disease prevention, health management, and a focus on prophylaxis instead of therapy have played a main role. This paradigm shift is due to the use of epidemiological science in livestock diseases and thus evaluation of problems at herd rather than individual animal level (15). Therefore, the veterinarian has become an essential part of this herd management, and his/her role has evolved away from purely curative work toward advisory work (8, 16). VHHM, in its position of independence from interests and finances, offers the possibility of a regular farm audit, without immediate negative consequence for premium reduction or similar, as, for example, is the case with quality management milk (QM) or cross-compliance inspections (CC) (17, 18). Peer collaboration between farmer and veterinarian in the interest of the farmer to reach the set targets can have demonstrable impact on performance (19, 20), as well as on animal health and welfare (6, 21), especially as the former requires the latter. As a study proved, information for disease prevention and thus optimized farm management is generally provided by the veterinarian through a VHHM program, but eventually depends on the farmer to implement suggestions (22, 23). It was also shown that the extent of VHHM participation varies considerably. Some farmers understand this to mean pregnancy checks, whereas others make use of the greatest possible range of services offered by the veterinarian, such as udder or young stock health (24–26). Regardless of the participation rate, the literature is controversial on the economic benefit of such VHHM programs. Two studies reported better farm performance and thus financial benefits with participation (27, 28), and another study showed an effect, although that disappeared after termination of the VHHM program (29). However, other research could not show a significant effect during participation (30). Another consideration is the cost and time of a veterinary farm visit (21, 28, 31–33), whereby the goal should be that the progress pays off the (cost) effort.

As study results showed before (26), the calculated overall satisfaction with VHHM was normally distributed and was rated, on average, as “good,” which was observed in other studies before (34, 35). The individual scope of VHHM was assessed by components with associated subquestions (e.g., VHHM component “udder health” included subquestions “evaluation of herd performance data,” “milk sampling,” “assessment of parlor routine”). The agreement to all VHHM components would have resulted in a scope of 100%. The average level of participation in our study was 36% and indicated that VHHM is still too focused on a few areas rather than taking a multidisciplinary approach. VHHM satisfaction correlated with scope of VHHM, and a possible reason for that is that a holistic herd management keeps several aspects overlooked.

The aim of the study was to describe the current practice of VHHM on German dairy farms. In the first part of the study, dairy farmers' attitude toward and satisfaction with VHHM have been displayed to enable future veterinarians to offer more adequate tailored concepts (26). The aim of the present article was to explore the associations between farm performance and the participation in and the satisfaction with VHHM. It was expected that in the group of VHHM farms, higher satisfaction with the veterinarian and the VHHM program would result in optimized farm performance parameters.

## Materials and Methods

### Study Design

This cross-sectional study was conducted from November 1 to December 31 of 2020 via the online survey tool LimeSurvey^®^ (LimeSurvey GmbH, Hamburg, Germany).

### Questionnaire Design

The questionnaire with a total of 123 questions was created based on a study from the Netherlands (36). Depending on answers given in the first few questions, the further questions differed in amount and content between VHHM participants and non-VHHM participants. Therefore, the time frame needed to answer the questionnaire was evaluated by a two-step pretest and took 20 min for VHHM participants and 12 min for non-VHHM participants.

The included questions were closed single-choice questions, questions with 5-point Likert scales, open-ended questions, and ranking questions. Page 1 contained details of goal and process of the survey and a privacy notice from the conducting Institute of Freie Universität Berlin as well as a data processing consent form was added.

While the first block included questions on general farm data, the second section covered available workforce on the farm. Further on, participants were asked about their subjective definition of VHHM, as participants were intentionally not given a definition of VHHM to prevent bias. Therefore, the classification for participation in a VHHM was left to the participants themselves. This question was followed by a key question on individual participation in a VHHM program, which decided about the further questions asked: Those who denied participation in a VHHM program at the time of the survey followed up with questions about possible potential on their farm and their willingness to pay for veterinary consultation while participants who stated to receive VHHM support on their farm were asked about the detailed design of the service. Eventually, questions regarding the demographics of the participants were asked.

A two-phase pretesting consisted of two phases: Phase I, where three dairy farmers were asked to sample the questionnaire in presence of the first author for understanding of questions and answer options. Subsequently, a few questions were adapted in terms of understanding. In phase II, three different dairy farmers were selected to complete the online format of the questionnaire without prior explanation, whereas the first author recorded the time required. Comprehension problems no longer existed in this phase, but a few questions were shortened, so the time limit of the survey was realistic.

### Participants

The voluntary participation in the survey was only possible online, and no regional limitations were given. To disseminate the study among the target group, farmers' associations were asked to spread the link among their members (“Deutscher Bauernverband”: 18 associations led by the head association). Moreover, other farmers' associations were contacted by mail and asked for assistance (“Bundesverband der Maschinenringe e.V.” (with all subassociations), “Bund Deutscher Milchviehhalter e.V.,” “Deutscher Raiffeisenverband e.V.,” “Bund der deutschen Landjugend e.V.”). The six largest dairy associations as well as organic associations were likewise included. The willingness of support was given among the sought-after as most replied on first contact.

### Statistical Analysis

The data were imported into IBM SPSS Statistics 27 (SPSS for Windows, IBM^®^, Armonk NY, USA) for descriptive data analysis and additionally transferred to R Studio [version 4.0.3. “Bunny-Wunnies Freak Out”; R Core Team, 2020 using the packages “dplyr” (37); “car” (38), “desctools” (39), “lmertest” (40), and “corrplot” (41)] for further analysis.

The amount that did not complete the survey and exited before the limit we set was due to analytical reasons: answered questionnaires to at least page 3, including questions of general farm data, available labor force and relative importance of VHHM definition, animal health decision-making, and satisfaction with veterinarian, were included in the analysis. The replies included were examined for duplication using the SPSS function and then subjected to further plausibility checks. No duplicates were identified, missing values were not filled in, and implausible values were removed but not replaced. Frequency tables were created for categorical variables. Continuous variables were checked for normal distribution using histograms and boxplots. To test the stochastic independence of the variables, the Wilcoxon rank test was performed in the part of the descriptive farm data.

The mean values of the variable blocks “advantages,” “disadvantages,” “fulfillment of expectations by vet,” “cooperation with vet,” and “improvements of VHHM” (matrix questions with Likert scale) were used to calculate a new variable (mean value of all equally weighted Likert scales), describing the overall satisfaction with the current VHHM.

Furthermore, to determine the scope of a farm's VHHM program, each VHHM component was scored based on its subquestions (e.g., VHHM component “udder health” included the subquestions “evaluation of herd performance data,” “milk sampling,” “assessment of parlor routine”). Components were weighted equally, and according to the number, the weight of individual subquestions was adjusted. Agreement on all subquestions of all VHHM components would have resulted in a scope of 100%.

Correlation coefficients were used to determine undirected correlations, and in case of normally distributed, metrically scaled data, Bravais-Pearson method was used, whereas, if one variable was at least ordinally scaled, Spearman rank correlation coefficient was calculated.

Regression models were developed using R Studio. First, single-factorial linear regression models were calculated with the performance parameters (dependent variable) and participation in VHHM (influencing variable). For those models where the participation had a *p* <0.2 and showed normal distribution of residuals, a multifactorial model including relevant confounders was calculated. Therefore, the impact of the participation in VHHM on performance parameter was assessed adjusting for the confounders herd size, region, breed, conventional or organic farming, husbandry system, and staffing ratio. Because of multiple testing, a Bonferroni correction was applied leading to a level of significance of 0.05/5 = 0.01.

To assess if a higher satisfaction or higher scope with VHHM or more frequent visits were associated with a higher 305-day milk yield (305dMY) or a lower age at first calving (AFC), multifactorial linear regression models were calculated including the mentioned variables and confounders (herd size, conventional or organic farming, husbandry system).

The requirements regarding linearity, homoscedasticity, and multicollinearity were tested using the QQ plot of residuals for visual inspection of normal distribution, the Breusch-Pagan test, and Cramer *V* and variation inflation coefficients, respectively.

## Results

### All Participants

With 57,322 registered German dairy farms at the time of evaluation and 216 evaluable questionnaires, the response rate results in 0.38%. Two hundred sixteen of 434 questionnaires were either fully (166×) or partially (50×) completed and were included in the analysis, whereas the remaining 218 did not complete the survey and were excluded from evaluation. Approximately half of the study participants (*n* = 106) participate in VHHM (Table 1). The VHHM farms kept higher numbers of animals (total stock of milk production including young stock) (mean = 491) compared with non-VHHM farms (mean = 360) (*p* = 0.0793). The mean 305dMY on VHHM farms differed by 1,218 kg compared with non-VHHM farms (10,195 vs. 8,977 kg; *p* < 0.0001). Mean AFC on VHHM farms was 26 months, whereas AFC on non-VHHM farms was 27 months (*p* = 0.0020). The mortality of cows in the period up to 60 days in milk (MORT60DIM) was slightly higher on VHHM farms (mean = 5.47%) than on non-VHHM farms (mean = 4.89%) (*p* = 0.2527). VHHM farms had a mean staffing ratio of 94 animals/staff member, compared with non-VHHM farms with a mean of 84 animals/staff member (*p* = 0.3507).

**Table 1:** Descriptive data of farm characteristics.

		**VHHM participation**	
		**Yes**	**No**
Total no. of animals for milk production (including offspring)	n	106	110
	25%	150	120
	Mean	491	360
	Median	243	200
	75%	479	400
	SD	978	450
	*p*-value		0.0793
No. of animals: lactating/dry (productive part of herd—in lactation or in dry period)	*n*	106	110
	25%	76	65
	Mean	217	191
	Median	130	105
	75%	270	238
	SD	225	228
	*p*-value		0.0869
305-day milk yield in kg	*n*	106	110
	25%	9,500	8,000
	Mean	10,195	8,977
	Median	10,399	9,120
	75%	11,200	10,100
	SD	1,524	1,793
	*p*-value		<0.0001
Energy corrected milk in kg	*n*	105	107
	25%	21.18	17.73
	Mean	22.58	20.20
	Median	23.09	21.05
	75%	24.56	23.13
	SD	2.93	4.16
	*p*-value		<0.0001
Bulk tank somatic cell count in thousands/mL (average of last 2 months)	*n*	106	110
	25%	125.50	130.50
	Mean	176.23	179.16
	Median	165.50	178.25
	75%	226.00	224.00
	SD	69.47	78.06
	*p*-value		0.5434
Age at first calving in months	*n*	106	110
	25%	25	25
	Mean	26	27
	Median	26	26
	75%	27	28
	SD	2	3
	*p*-value		0.0020
Replacement rate in%	*n*	82	76
	25%	23	20
	Mean	28	27
	Median	28	28
	75%	32	34
	SD	6	9
	*p*-value		0.9763
Mortality <60 days in milk	*n*	59	56
	25%	1.00	0
	Mean	5.47	4.89
	Median	2.00	2.50
	75%	8.00	5.00
	SD	6.82	6.57
	*p*-value		0.2527
Staffing ratio: total stock (no. of animals/staff)	*n*	106	110
	25%	54.59	48.00
	Mean	93.65	84.19
	Median	80.24	72.86
	75%	100.00	105.00
	SD	82.24	67.05
	*p*-value		0.3507
Staffing ratio: lactating/dry (no. of animals/staff) (no. of animals of productive part of herd—in lactation or in dry period—per staff)	*n*	106	110
	25%	30.00	26.00
	Mean	48.05	45.13
	Median	43.07	38.13
	75%	54.67	55.96
	SD	35.80	36.42
	*p*-value		0.3401

*Survey among German dairy farmers on VHHM participation (n = 216). p-values were calculated using the Wilcoxon rank test*.

More than three-quarters of the VHHM farms “always/often” discussed decisions with their veterinarian, whereas a little over half of the non-VHHM farms did so (Table 2). The comparison of means revealed that VHHM farms reporting taking important decisions “always” with their veterinarian had numerically the highest 305dMY compared with farmers taking decisions less frequently with their veterinarian. VHHM farms that “always” discussed decisions had numerically the lowest bulk tank somatic cell count (BTSCC). The replacement rate (RR) reached the numerical maximum value within those who “occasionally” made decisions with the veterinarian, regardless of VHHM participation. The AFC was numerically lowest within the VHHM farms that answered “always” with 25 months.

**Table 2:** Descriptive data of decision-making with veterinarian.

**Comparison of means: decision-making with vet**							
	**VHHM**	**305dMY** **(kg)**	**ECM** **(%)**	**BTSCC** **(×1,000/mL)**	**MORT60DIM** **(%)**	**RR** **(%)**	**AFC** **(months)**
Always	Yes	*n* = 21 10,639	*n* = 20 23.69	*n* = 21 155	*n* = 11 7.55	*n* = 17 28.12	*n* = 21 25.0
	No	*n* = 15 8,916	*n* = 15 20.38	*n* = 15 201	*n* = 9 5.11	*n* = 9 27.33	*n* = 15 26.7
Often	Yes	*n* = 63 10,297	*n* = 63 22.63	*n* = 63 183	*n* = 36 5.36	*n* = 51 27.76	*n* = 63 26.1
	No	*n* = 47 8,732	*n* = 47 19.94	*n* = 48 171	*n* = 21 5.14	*n* = 28 27.14	*n* = 48 27.4
Occasionally	Yes	*n* = 18 9,844	*n* = 18 21.93	*n* = 18 171	*n* = 9 4.22	*n* = 11 29.00	*n* = 18 26.1
	No	*n* = 30 9,022	*n* = 30 20.02	*n* = 31 179	*n* = 16 5.50	*n* = 24 29.71	*n* = 31 27.5
Rare	Yes	*n* = 2 7,464	*n* = 2 19.66	*n* = 2 161	*n* = 1 1.00	*n* = 2 20.00	*n* = 2 26.5
	No	*n* = 14 9,943	*n* = 14 21.58	*n* = 15 184	*n* = 10 3.20	*n* = 14 24.43	*n* = 15 26.1
Never	Yes	*n* = 2 8,200	*n* = 2 18.68	n = 2 233	*n* = 2 4.00	*n* = 1 24.00	*n* = 2 28.5
	No	*n* = 1 6,500	*n* = 1 15.65	*n* = 1 178	*n* = 0 —	*n* = 1 20.00	*n* = 1 26.0

*Survey among German dairy farmers on VHHM participation (n = 216)*.

Results in Table 3 demonstrate that more than one-third of the VHHM farms and approximately one-quarter of the non-VHHM farms rated the question about satisfaction with their veterinarian with “very good.” Numerically lowest 305dMY had non-VHHM farms that selected “sufficient” or “unsatisfactory. Analysis showed that non-VHHM farms that indicated “very good” had the numerically lowest BTSCC values. Non-VHHM farms reporting “sufficient” or “unsatisfactory” had the numerically highest BTSCC values, respectively. Regardless of the level of satisfaction with the veterinarian, the numerical values for AFC were between 25 and 26 months for VHHM farms and between 26 to 28 months for non-VHHM farms.

**Table 3:** Descriptive data of satisfaction with veterinarian.

**Comparison of means: satisfaction with vet**							
	**VHHM**	**305dMY** **(kg)**	**ECM** **(%)**	**BTSCC** **(×1,000/mL)**	**MORT60DIM** **(%)**	**RR** **(%)**	**AFC** **(months)**
Very good	Yes	*n* = 40 10,405	*n* = 40 23.11	*n* = 40 173	*n* = 25 5.08	*n* = 31 26.06	*n* = 40 25.8
	No	*n* = 30 8,433	*n* = 31 19.40	*n* = 31 168	*n* = 19 4.68	*n* = 20 25.20	*n* = 31 27.1
Good	Yes	*n* = 45 10,149	*n* = 44 22.39	*n* = 45 183	*n* = 22 6.50	*n* = 34 28.56	*n* = 45 26.1
	No	*n* = 52 9,297	*n* = 52 20.78	*n* = 45 172	*n* = 26 5.54	*n* = 39 28.15	*n* = 54 26.8
Satisfactory	Yes	*n* = 14 9,762	*n* = 14 21.58	*n* = 14 169	*n* = 10 4.10	*n* = 11 28.82	*n* = 14 25.6
	No	*n* = 16 9,464	*n* = 15 20.77	*n* = 16 195	*n* = 4 6.00	*n* = 10 29.70	*n* = 16 28.4
Sufficient	Yes	*n* = 4 8,950	*n* = 4 20.10	*n* = 4 157	*n* = 1 10.00	*n* = 4 35.00	*n* = 4 25.5
	No	*n* = 5 8,070	*n* = 5 18.01	*n* = 5 240	*n* = 4 3.25	*n* = 3 19.67	*n* = 5 26.6
Unsatisfactory	Yes	*n* = 3 11,767	*n* = 3 26.30	*n* = 3 175	*n* = 1 2.00	*n* = 2 20.50	*n* = 3 26.7
	No	*n* = 4 8,063	*n* = 4 19.37	*n* = 4 218	*n* = 3 1.33	*n* = 4 30.75	*n* = 4 28.0

*Survey among German dairy farmers on VHHM participation (n = 216)*.

The mean comparison of the ranking question about their relative importance of the term “Veterinary Herd Health Management” (Table 4) showed that the numerically highest 305dMY was reached by the subgroup that ranked “herd data/strategy planning/economy” first. VHHM farms, which considered “problem-solving” in their definition of VHHM, had numerically lowest 305dMY.

**Table 4:** Descriptive data of relative importance of subjective definition of VHHM.

**Comparison of means: definition of VHHM** ***—*** **rank 1**							
	**VHHM**	**305dMY** **(kg)**	**ECM** **(%)**	**BTSCC** **(×1,000/mL)**	**MORT60DIM** **(%)**	**RR** **(%)**	**AFC** **(months)**
Pregnancy checks and support in reproduction	Yes	*n* = 53 10,166	*n* = 52 22.60	n = 53 170	*n* = 28 4.61	*n* = 39 27.74	*n* = 53 25.9
	No	*n* = 43 9,325	*n* = 43 20.96	*n* = 44 171	*n* = 25 3.88	*n* = 29 27.00	*n* = 44 27.4
Problem solving	Yes	*n* = 33 10,213	*n* = 33 22.46	*n* = 33 194	*n* = 22 6.59	*n* = 27 27.44	*n* = 33 25.7
	No	*n* = 45 8,417	*n* = 43 19.29	*n* = 45 179	*n* = 21 4.76	*n* = 32 28.03	*n* = 45 27.2
Herd data/strategy planning/farm economy	Yes	*n* = 20 10,241	*n* = 20 22.73	n = 20 163	*n* = 9 5.44	n = 16 28.38	*n* = 20 26.3
	No	*n* = 19 9,513	*n* = 21 20.47	*n* = 21 197	*n* = 10 7.70	*n* = 15 26.73	*n* = 21 26.4

*Survey among German dairy farmers on VHHM participation (n = 216)*.

In single regression models, only 305dMY and AFC showed significant associations (*p* ≤ 0.001), and thus, multiple regressions were created for these variables. In multifactorial modeling for 305dMY, adjusted for herd size, region, breed, farm management, housing type, and staffing ratio, VHHM farms had a 660-kg higher 305dMY than non-VHHM farms (adjusted *R*^2^ = 0.6335) (Table 5). Moreover, AFC on VHHM farms was 0.8 months lower than on non-VHHM farms when adjusted for the confounders written above (*p* = 0.045).

**Table 5:** All participants: multiple regression models.

**Multiple regression model: 30** ***5d*** **MY (Analysis of dependent variable “305dMY” with influencing variables)**						
**305dMY ~ participation in VHHM + herd size: lactating/dry + region + breed + conventional/organic + husbandry system + staffing ratio: lactating/dry**						
Degrees of freedom = 159				*p* = 0.001		
Multiple *R*^2^ = 0.6552				Adjusted *R*^2^ = 0.6335		
	**Minimum**	**25%**	**Median**	**75%**	**Maximum**	**Standard error**
Residuals	−1992.5	−768.2	−51.0	671.0	2953.2	1,102
			**Estimate**	**Standard error**	***t*** **-value**	***p*** **-value**
305dMY (VHHM + region north + Holstein + conventional + free stall + staffing ratio)			10,305.91	267.15	38.58	<0.001
No VHHM			−659.91	182.68	−3.61	<0.001
Herd size: lactating/dry			1.99	0.51	3.95	<0.001
Region east			−542.89	331.70	−1.64	0.104
Region south			937.53	341.88	2.74	0.007
Region west			−11.19	210.89	−0.05	0.958
No Holstein			−1,938.52	293.02	−6.62	<0.001
Organic			−1,760.57	310.51	−5.67	<0.001
Free stall + pasture			−709.12	211.84	−3.35	0.001
Tie stall (+/– pasture)			−1,171.60	449.39	−2.61	0.010
Staffing ratio: lactating/dry			1.03	2.40	0.43	0.668
**Multiple regression model** ***: A*** **FC (Analysis of depending variable “AFC” with influencing variables)**						
**AFC ~ participation in VHHM + herd size: lactating**/**dry animals + region + breed + conventional**/**organic + staffing ratio: lactating**/**dry animals**						
Degrees of freedom = 164				*p* = 0.001		
Multiple *R*^2^ = 0.3476				Adjusted *R*^2^ = 0.3158		
	**Minimum**	**25%**	**Median**	**75%**	**Maximum**	**Standard error**
Residuals	−5.03	−1.36	−0.06	1.17	17.87	2.43
			**Estimate**	**Standard error**	***t*** **-value**	***p*** **-value**
AFC (VHHM + herd size + region north + Holstein + conventional + staffing ratio)			25.97	0.54	48.55	<0.001
No VHHM			0.80	0.40	2.02	0.045
Herd size: lactating/dry			−0.002	0.001	−1.74	0.084
Region east			−0.07	0.73	−0.10	0.922
Region south			−1.00	0.74	−1.35	0.179
Region west			0.83	0.46	1.80	0.073
No Holstein			2.38	0.63	3.76	<0.001
Organic			2.21	0.63	3.53	<0.001
Staffing ratio: lactating/dry			−0.01	0.01	−1.56	0.120

*Survey among German dairy farmers on VHHM participation (n = 216)*.

### Participants in VHHM

Table 6 shows that, in the single regression analysis of the variables (VHHM scope, VHHM satisfaction, visit frequency) with the performance data (305dMY, AFC, BTSCC), only a significant correlation between 305dMY and VHHM scope could be found (*p* = 0.039). With each percent more VHHM scope the 305dMY raised by 18 kg. However, multifactorial modeling of the variables visit frequency, VHHM satisfaction, VHHM scope, herd size, farm management, and housing type showed no significant impact on the 305dMY and the AFC.

**Table 6:** VHHM participants: single/multiple regression models survey among German dairy farmers on VHHM participation (*n* = 216).

**Single regression models**						
***p*** **Value**	**BTSCC**		**AFC**		**305dMY**	
VHHM scope	0.717		0.066		0.039	
VHHM satisfaction	0.52		0.378		0.13	
Visit frequency	0.943		0.321		0.538	
**Single regression model: 30** ***5d*** **MY**						
**305dMY ~ VHHM scope (Analysis of depending variable “305dMY” with influencing variable “scope”)**						
Degrees of freedom = 71				*p* = 0.03916		
Multiple *R*^2^ = 0.05855				Adjusted *R*^2^ = 0.04529		
	**Minimum**	**25%**	**Median**	**75%**	**Maximum**	**Standard error**
Residuals	−161.74	−47.74	−9.24	31.26	186.76	70.98
			**Estimate**	**Standard error**	***t-*** **value**	***p*** **-value**
305dMY (VHHM scope)			9,597.90	361.08	26.58	<0.001
VHHM scope			18.34	8.73	2.10	0.0392
**Multiple regression model: 30** ***5d*** **MY**						
**305dMY ~ visit frequency + VHHM satisfaction + VHHM scope + herd size + conventional**/**organic + husbandry system (Analysis of depending variable “305dMY” with influencing variables)**						
Degrees of freedom = 47				*p* = 0.0002413		
Multiple *R*^2^ = 0.4489				Adjusted *R*^2^ = 0.3551		
	**Minimum**	**25%**	**Median**	**75%**	**Maximum**	**Standard error**
Residuals	−2,413.63	−790.83	−70.86	839.75	2,055.16	1,163
			**Estimate**	**Standard error**	***t-*** **value**	***p*** **-value**
305dMY (visit <1 × /month + VHHM satisfaction + VHHM scope + herd size + conventional + free stall)			9,600.81	1,148.71	8.36	<0.001
Visit 1 × /month			−61.99	371.33	−0.17	0.868
Visit > 1 × /month			−344.31	442.22	−0.78	0.440
VHHM satisfaction			260.17	454.55	0.57	0.570
VHHM scope			13.61	9.23	1.48	0.147
Herd size			1.15	0.76	1.51	0.138
Organic			−2,078.33	726.54	−2.86	0.006
Free stall + pasture			−882.12	401.62	−2.20	0.033
Tie stall (+/– pasture)			−2,778.18	896.40	−3.10	0.003
**Multiple regression model** ***: A*** **FC**						
**AFC ~ visit frequency + VHHM satisfaction + VHHM scope + herd size + conventional**/**organic + husbandry system (Analysis of depending variable “AFC” with influencing variables)**						
Degrees of freedom = 47				*p* = 1.423^−05^		
Multiple *R*^2^ = 0.5197				Adjusted *R*^2^ = 0.438		
	**Minimum**	**25%**	**Median**	**75%**	**Maximum**	**Standard error**
Residuals	−3.11	−1.07	−0.19	0.97	2.75	1.56
			**Estimate**	**Standard error**	***t-*** **value**	***p*** **-value**
AFC (visit <1 × /month + VHHM satisfaction + VHHM scope + herd size + conventional + free stall)			28.09	1.54	18.29	<0.001
Visit 1 × /month			−0.82	0.50	−1.65	0.105
Visit > 1 × /month			−0.49	0.59	−0.83	0.410
VHHM satisfaction			−0.56	0.61	−0.92	0.361
VHHM scope			−0.02	0.01	−1.50	0.141
Herd size			−0.002	0.001	−2.05	0.046
Organic			5.32	0.97	5.48	<0.001
Free stall + pasture			0.11	0.54	0.21	0.834
Tie stall (+/– pasture)			1.66	1.20	1.39	0.173

## Discussion

The hypothesis tested was the presence of an association between farm performance and participation in VHHM. The results indicate a statistically significant association between participation and milk yield as well as AFC. However, no significant associations were detected between the different services of VHHM and performance.

According to the Federal Statistical Office of Germany, 57,322 dairy farms were registered in the entire country in 2020. Thus, the participating 216 farms represented 0.38% of dairy farms in Germany (2). As a low percentage of the target population could be reached, results may be biased, and this issue can be found in previous studies (36). Validity of results is influenced by the sample size and several other factors, as also analyzed in part 1 of the study (26). As the survey was exclusively accessible online, selection bias was possibly present as it significantly contributes to a reduced range and thus smaller number of possible participants. Online recruitment only targeted dairy farms with email addresses and membership in association mailing lists or access to social media. An equally important factor was the participants' personal affinity for online media and their motivation on the relevant topic (42). A former study showed a shift of participants toward larger farms (24), which is also evident from the discrepancy of the mean number of cows per farm in our study and the 2020 nationwide average of dairy cows (2, 3). However, the aim of this part of the study was not to assess a representative status but to assess a possible relationship between the participation in VHHM and farm performance.

A weakness of the study is undoubtedly the fact that the data were collected by questionnaire. There were no farm visits, so we deliberately omitted to ask about health-related factors of the animals to prevent gross misrepresentation. Despite that participation was explicitly voluntary and anonymous, survey participants generally tend to give a distorted picture of themselves (43). Especially the results of performance parameters could deviate from reality. To prevent this aspect, we asked in the introduction to take data straight from the current MLP (provided participants with the exact page and field reference in this document to find the data). Because of this indication and the guaranteed anonymity, we assume that the information provided was mostly valid. Furthermore, the division into VHHM participants and non-VHHM participants was based solely on the participants' self-assessment, as no definition of VHHM was deliberately given, in order to prevent inhibited participation or misperceptions. This may have resulted to farmers having been assigned to the wrong group. Also, this is a possible explanation for the big amount of VHHM participants whose subjective definition leaned toward pregnancy checks, which does not symbolize the all-encompassing attempt (44).

### All Participants

The fact that VHHM farms were significantly larger in animal numbers is congruent with a previous study (24). However, it is conceivable that farms with more animals are more interested in current issues and more likely to participate in surveys (45). Another consideration may be that either farms with greater animal numbers are more likely to participate in VHHM, or the structure gained through VHHM participation allows an expansion, but that remains unclear in the final extent.

Moreover, in multiple regression, VHHM farms had a statistically significantly higher 305dMY of more than 600 kg adjusted for region, breed, farm management, housing type, and staffing ratio. Previous studies showed that VHHM-supported farms have significantly higher milk yields than non-VHHM farms (8, 24, 28, 33). The reasons for that can be manifold, as described herein, such as the appreciation of benefits of prophylaxis and a strategic approach.

In addition, a 0.8-month lower AFC on the VHHM farms also suggests better farm management: The AFC has been shown to be influenced by young stock rearing practices, for example, the amount and kind (no waste milk) of milk fed preweaning (46). Several studies show that heifer growth is essentially influenced by the feeding management at calf age, and rearing conditions have an immense effect on the potential of the future dairy cow (47, 48). It would be conceivable that this reflects the merit of VHHM on those farms.

Given the results of the performance parameters, the approach described in a previous study (15) fits: The main characteristic of VHHM is the integrated farm assessment based on valid, collected data and in consideration of economic interests. This approach, they argue, serves to prevent disease and to increase performance. The effect of increased overall performance with VHHM participation has additionally been demonstrated in other studies (28). Nevertheless, this key finding offers potential for further research to determine whether increased performance is a cause or effect of VHHM participation.

For the higher MORT60DIM on VHHM farms, no significant correlation could be shown further on. Still, it could be a consequence of higher milk yields and the purported occurrence of production diseases (11, 49–51) during the critical transition period and subsequently increased involuntary cull of animals (52, 53). Data from Israel explain the occurrence of these diseases through a deficient management and a reduction of said after an intensified VHHM program (50). For intensified dairy farming, it is even more important to put animal welfare at the forefront. Intensification that ignores animal welfare is not a promising or sustainable approach to future farming. The veterinarian is obliged to emphasize this in the interest of the animals within the framework of a VHHM program, which in turn will pay off (54).

The staffing ratio, relating to the total number of animals, might reflect a more intensive form of farming. With 94 animals per staff member on VHHM farms and 84 animals per staff member on non-VHHM farms, the results of this study are in the range of the staffing ratio of US farms, where values between 80 and 100 animals per employee have been described (55–57). Regardless of participation in VHHM, our study found that with each additional animal per staff member, BTSCC increased by 300 cells/mL of milk. Relatively less staff may lead to a less optimized parlor routine and/or routine of bedding maintenance, which implies advantages of a closer ratio.

The more intensive collaboration between veterinarian and farmer in decision-making on VHHM farms is also reflected in the results of previous studies (58). Indirectly, the scope of collaboration appears to be related to farm performance, as farms that consult more frequently with their veterinarian and farms that are more satisfied with their veterinarian both performed better in the mean comparison. As the veterinarian counts as one of the most important advisors to the farm (24, 59), the relationship with the veterinarian is an important contributor to success (28).

Furthermore, survey participants who understood that VHHM was “herd data/strategy planning/economy,” that is, the most encompassing management approach among the definitions given, had the highest farm performance parameters, regardless of their participation in VHHM. This contrasted with the non-VHHM participants, who defined VHHM primarily as problem-solving and had the lowest milk performance of all. The once exclusively therapeutic task of a veterinarian over the years has been increasingly replaced by a disease prophylactic task, so that the commonly known “firefighting veterinarian” is replaced (8, 21).

### Participants in VHHM

Certain associations, such as the presence of an effect of VHHM scope, VHHM satisfaction, and frequency of visits or support in a specific area (like udder health) combined with performance parameters, could not be verified in our study. The reason for this could be that the sample size was too small, at least for certain combinations of risk factors. This result may give reason to believe that the extent and design of a VHHM appear to be secondary to farm performance data and are well-worthy subject to further research.

## Conclusion

The study outlined that participation in a VHHM program showed significant differences in the performance benchmarks 305dMY and AFC. Consequently, it is important for a farm to have VHHM participation theoretically. Within the VHHM participants, however, the detailed extent of herd management did not play a role in this present study. Thus, it can be assumed from this research that a participation in VHHM may lead to a better performance of the dairy herd. Regardless of the new legal situation, the results of the present study may contribute to higher intrinsic motivation among dairy producers to participate in a VHHM program.

## Data Availability Statement

The raw data supporting the conclusions of this article will be made available by the authors, without undue reservation.

## Ethics Statement

In this study, no personal or sensitive data was collected. Participation was voluntary and anonymous. Before starting the questionnaire, participants perceived detailed information about the aims of the study and how the data were evaluated. Consent needed to be actively given by each participant. For no personal rights or any German and European data protection laws could be violated, we refrained from receiving approval from an Ethics Committee.

## Author Contributions

JR conceived and designed the study, developed the theoretical framework, and implemented it in a preliminary model and questionnaire. Statistical preliminary considerations and statistical analyses were performed in close cooperation with RM and KJ. JR drafted and revised the manuscript. RM, KM, and CT-R supervised and supported the project at each point of the development, conduction, statistical evaluation, and during the paper-writing process. All authors contributed to the article and approved the submitted version.

## Conflict of Interest

The authors declare that the research was conducted in the absence of any commercial or financial relationships that could be construed as a potential conflict of interest.

## Publisher's Note

All claims expressed in this article are solely those of the authors and do not necessarily represent those of their affiliated organizations, or those of the publisher, the editors and the reviewers. Any product that may be evaluated in this article, or claim that may be made by its manufacturer, is not guaranteed or endorsed by the publisher.

## Abbreviations

AFC: age at first calving
AMS: automatic milking system
BTSCC: bulk tank somatic cell count
CC: cross compliance
DIM: days in milk
EU: European Union
MLP: German DHI testing
MORT60DIM: mortality of cows in the period up to 60 days in milk
QM: German Quality Management Milk
RR: replacement rate
VHHM: Veterinary Herd Health Management
305dMY: 305-day milk yield.
