# Supplementary material for: Impact of Veterinary Herd Health Management on German Dairy Farms: Effect of Participation on Farm Performance
Source: Front Vet Sci. 2022 Apr 7;9:841405. doi: 10.3389/fvets.2022.841405 (PMC9021589; doi:10.3389/fvets.2022.841405)
Supplement: Supplementary file 1 [file Data_Sheet_1.PDF]

Nutzen der Integrierten Tierärztlichen Bestandsbetreuung (ITB) in deutschen Milchviehbetrieben

Umfrage für die Promotion von Tierärztin Jenny Ries, Freie Universität Berlin

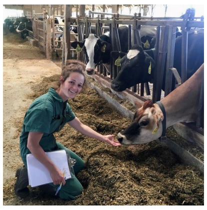

Liebe Landwirtinnen, liebe Landwirte,

im Rahmen meiner Doktorarbeit am Institut für Veterinärepidemiologie und Biometrie, Freie Universität Berlin beschäftigen wir uns mit der derzeitigen Verbreitung und Gestaltung von Integrierter Tierärztlicher Bestandsbetreuung (ITB).

Dafür möchten wir mit diesem Fragebogen sowohl die Zusammenarbeit zwischen Tierarztpraxis und Betrieb als auch Veränderungswünsche seitens des Betriebes genauer unter die Lupe nehmen und so den Nutzen von ITB in deutschen Milchviehbetrieben untersuchen. Zudem wird der Zusammenhang zwischen Mitarbeiteranzahl und -qualifikation auf die Tiergesundheit und die betriebliche Situation untersucht.

Über Ihre Teilnahmebereitschaft freuen wir uns sehr!

Zur Vorbereitung können Sie Ihre aktuellste Betriebsübersicht (letzte MLP, Roboterauswertung,...) bereitlegen, um so den Teil der Fragen schnell und problemlos auszufüllen.

Die Beantwortung des Fragebogens wird etwa 15-20 Minuten in Anspruch nehmen.

Dank des großzügigen Sponsorings haben Sie die Möglichkeit der Verlosung von attraktiven Zeitschriften-Abonnements (zeitlich befristet: agrarheute, Elite, Landwirt, topagrar). Die Teilnahme hieran wird im Anschluss erfragt, ist nicht verpflichtend und selbstverständlich unabhängig von Ihren Antworten.

Herzlichen Dank für Ihre Zeit!

Jenny Ries
(Tierärztin und Doktorandin, Institut für Veterinärepidemiologie und Biometrie, FU Berlin)

Bei Fragen oder Anmerkungen wenden Sie sich gerne an Frau Dr. Merle im Institut für Veterinärepidemiologie und Biometrie, FU Berlin (roswitha.merle@fu-berlin.de, Tel. 030 838 75096)

https://www.vetmed.fu-berlin.de/einrichtungen/institute/we16/index.html (https://www.vetmed.fu-berlin.de/einrichtungen/institute/we16/index.html)

In dieser Umfrage sind 124 Fragen enthalten.

Welche Art von Betrieb bewirtschaften Sie? *

Bitte wählen Sie nur eine der folgenden Antworten aus:

- ◯ reiner Milchviehbetrieb
- ◯ gemischter Betrieb (Milchvieh + sonstige Nutzung)
- ◯ kein Milchviehbetrieb

Gesamtzahl an Bestandstieren, die für die Milchproduktion gehalten werden, inkl. Nachzucht: *

❗ In diesem Feld darf nur ein ganzzahliger Wert eingetragen werden.

Bitte geben Sie Ihre Antwort hier ein:

[ ]

Tiere

Anzahl an laktierenden und trockenstehenden Kühen: *

❗

❗ In diesem Feld darf nur ein ganzzahliger Wert eingetragen werden.

Bitte geben Sie Ihre Antwort hier ein:

[ ]

Kühe

Bitte nennen Sie die Gesamttierzahl von laktierenden und trockenstehenden Gruppen zusammengerechnet.

(z.B. in der MLP zu finden unter "Tagesleistungen - Kühe (Stall/ geprüft)", Seite 1)

Welche Milchviehrasse wird vorwiegend gehalten? *

Bitte wählen Sie nur eine der folgenden Antworten aus:

- ◯ Schwarzbunte Deutsche Holstein
- ◯ Rotbunte Deutsche Holstein
- ◯ Fleckvieh
- ◯ Braunvieh
- ◯ Jersey
- ◯ Sonstiges ____________

Wie bewirtschaften Sie Ihren Betrieb? *

Bitte wählen Sie nur eine der folgenden Antworten aus:

- ◯ konventionell
- ◯ ökologisch ("Bio")

Wie werden die laktierenden Tiere auf Ihrem Betrieb gehalten? *

Bitte wählen Sie nur eine der folgenden Antworten aus:

- ◯ Laufstallhaltung - ohne Auslauf
- ◯ Laufstallhaltung - mit Laufhof
- ◯ Laufstallhaltung - mit Weideauslauf
- ◯ Anbindehaltung - ohne Auslauf
- ◯ Anbindehaltung - mit Weideauslauf

Weideauslauf = in Summe > 6 Monate à mindestens 6 Stunden täglich

Welche Boxenart haben Sie in Ihrer laktierenden Gruppe? *

Bitte wählen Sie nur eine der folgenden Antworten aus:

- ◯ Hochbox - mit Matratze (z.B. Späne, Stroh)
- ◯ Hochbox - ohne Matratze
- ◯ Tiefbox
- ◯ Tiefstreufläche
- ◯ Sonstiges ____________

Sind auf Ihrem Betrieb Automatische Melksysteme (AMS/ Melkroboter) im Einsatz? *

Bitte wählen Sie nur eine der folgenden Antworten aus:

- ◯ Ja
- ◯ Nein

Nehmen Sie an der monatlichen Milchleistungsprüfung (MLP) teil? *

Bitte wählen Sie nur eine der folgenden Antworten aus:

- ◯ Ja
- ◯ Nein

Durchschnittliche Milchmenge pro Tier und Jahr Ihrer Herde? [in kg] *

❗ Ihre Antwort muss mindestens 1000 sein.
❗ In diesem Feld darf nur ein ganzzahliger Wert eingetragen werden.
Bitte geben Sie Ihre Antwort hier ein:

____
kg

(z.B. in der MLP zu finden unter "Gleitender Durchschnitt bis Monatsende - Betrieb, Mkg", Seite 1)

Wie hoch ist Ihre aktuelle durchschnittliche Milchmenge pro Kuh und Tag? [in kg] *

Bitte geben Sie Ihre Antwort hier ein:

kg

(z.B. in der MLP zu finden unter "Tagesleistungen - Milch-kg (gemolkene Kühe)", Seite 1)

Wie hoch ist Ihr aktueller Milchfettgehalt? [in %] *

Bitte geben Sie Ihre Antwort hier ein:

%

(z.B. in der MLP zu finden unter "Tagesleistungen - Fett%", Seite 1)

Wie hoch ist Ihr aktueller Milcheiweißgehalt? [in %] *

Bitte geben Sie Ihre Antwort hier ein:

%

(z.B. in der MLP zu finden unter "Tagesleistungen - Eiweiß %", Seite 1)

Tankzellzahl* der letzten zwei Prüfungen *

❶ Jede Antwort muss zwischen 10 und 999 sein
❶ Nur ganzzahlige Werte können in diese Felder eingegeben werden.
Bitte geben Sie Ihre Antwort(en) hier ein:

letzte Prüfung

vorletzte Prüfung

*Gehalt somatischer Zellen pro ml Milch im Sammeltank

(z.B. in der MLP zu finden unter "Tagesleistungen - Zellzahl (in 1000)", Seite 1)

Wie hoch ist der Abgang an Kühen unter 60 Tagen in Milch auf Ihrem Betrieb? [in %] *

❶ Wenn Sie 'Abgangsrate in %' auswählen, spezifizieren Sie bitte Ihre Auswahl im entsprechenden Textfeld.
❶ Nur Zahlen können in das 'Abgangsrate in %' begleitende Textfeld eingegeben werden.
Bitte wählen Sie nur eine der folgenden Antworten aus:

- ◯ keine Angabe
- ◯ Abgangsrate in %

Wie hoch ist die zuletzt berechnete Remontierungsrate in % auf Ihrem Betrieb? *

❶ Wenn Sie 'Remontierungsrate in %' auswählen, spezifizieren Sie bitte Ihre Auswahl im entsprechenden Textfeld.
❶ Nur Zahlen können in das 'Remontierungsrate in %' begleitende Textfeld eingegeben werden.
Bitte wählen Sie nur eine der folgenden Antworten aus:

- ◯ keine Angabe
- ◯ Remontierungsrate in %

Bitte geben Sie das durchschnittliche Erstkalbealter Ihrer Färsen an. *

❶ In diesem Feld darf nur ein ganzzahliger Wert eingetragen werden.
Bitte geben Sie Ihre Antwort hier ein:

Monate

(z.B. in der MLP zu finden unter "Fruchtbarkeitsinformation - Färsenbestand - EKA - Betrieb", Seite 3)

Wieviele Arbeitskräfte bewirtschaften Ihren Betrieb? *

❶ Nur ganzzahlige Werte können in diese Felder eingegeben werden.
Bitte geben Sie Ihre Antwort(en) hier ein:

Gesamtanzahl an Arbeitskräften (inkl. Sie selbst und Familienangehörigen)

davon Anzahl an familienangehörigen Arbeitskräften

Wieviele Personen arbeiten aktuell jeweils in den folgenden Anstellungsmodellen?

(Inkl. Sie selbst und Ihren Familienangehörigen. Bitte geben Sie die jeweilige Anzahl an.)

❶ Nur ganzzahlige Werte können in diese Felder eingegeben werden.
Bitte geben Sie Ihre Antwort(en) hier ein:

Vollzeit

Teilzeit

Mini-Job (450€ Basis)

Saisonarbeit

Haben Ihre Mitarbeiter Fachkompetenz im Bereich Milchviehwirtschaft und wenn ja, welche?

Bitte geben Sie jeweils die zutreffende Anzahl an Mitarbeitern ein.

Trifft auf einen Mitarbeiter mehr als eine der möglichen Antworten zu, wählen Sie bitte die "höherwertige" aus.

*

❶ Kommentieren wenn eine Antwort gewählt wird
❶ Wenn Sie 'sonstige Fachkompetenz' auswählen, spezifizieren Sie bitte Ihre Auswahl im entsprechenden Textfeld.
Bitte wählen Sie die zutreffenden Punkte aus und schreiben Sie einen Kommentar dazu:

- [ ] keine fachbezogene Ausbildung; wenig Erfahrung
- [ ] keine fachbezogene Ausbildung, aber mehrjährige Erfahrung (> 5 Jahre) durch Arbeit auf Milchviehbetrieben
- [ ] Landwirtschaftlicher Auszubildender
- [ ] Herdenmanagerkurs
- [ ] abgeschlossene landwirtschaftliche Ausbildung/ Studium

Sonstiges:

Hängen in Ihrem Betrieb aktuelle, schriftliche (ggf. fremdsprachige) Arbeitsanweisungen zu Alltagssituationen mit den Tieren aus? *

Bitte wählen Sie nur eine der folgenden Antworten aus:

- ◯ Ja
- ◯ Nein

Halten Sie regelmäßig Besprechungen mit den Betriebsangehörigen? *

Bitte wählen Sie nur eine der folgenden Antworten aus:

- ◯ Ja, mit allen
- ◯ Ja, aber nicht mit allen
- ◯ Nein

Nehmen Sie oder andere Mitarbeiter mind. 1x jährlich an externen Fortbildungen teil? *

Bitte wählen Sie nur eine der folgenden Antworten aus:

- ◯ Ja
- ◯ Nein

Beschäftigen Sie auf Ihrem Betrieb fremdsprachige Arbeitskräfte? *

Bitte wählen Sie nur eine der folgenden Antworten aus:

- ◯ Ja
- ◯ Nein

Aus welchen Ländern beschäftigen Sie fremdsprachige Mitarbeiter? *

Beantworten Sie diese Frage nur, wenn folgende Bedingungen erfüllt sind:
Antwort war 'Ja' bei Frage '24 [Bfremdspr]' (Beschäftigen Sie auf Ihrem Betrieb fremdsprachige Arbeitskräfte?)

❗ Bitte wählen Sie die zutreffenden Antworten aus:
Bitte wählen Sie alle zutreffenden Antworten aus:

- ☐ Polen
- ☐ Rumänien
- ☐ Bulgarien
- ☐ Ukraine
- ☐ Nord-/ ostafrikanische Länder
- ☐ Sonstiges: ____________

Verständigung auf dem Betrieb *

Beantworten Sie diese Frage nur, wenn folgende Bedingungen erfüllt sind:
Antwort war 'Ja' bei Frage '24 [Bfremdspr]' (Beschäftigen Sie auf Ihrem Betrieb fremdsprachige Arbeitskräfte?)

Bitte wählen Sie die zutreffende Antwort für jeden Punkt aus:

	**trifft voll zu (++)**	**trifft zu (+)**	**neutral (0)**	**trifft nicht zu (-)**	**trifft gar nicht zu (--)**
**Meine fremdsprachigen Mitarbeiter sprechen meinem Empfinden nach ausreichend Deutsch für die alltägliche Verständigung auf dem Betrieb.**	◯	◯	◯	◯	◯

In welchem Bereich setzen Sie fremdsprachige Mitarbeiter ein? *

Beantworten Sie diese Frage nur, wenn folgende Bedingungen erfüllt sind:
Antwort war 'Ja' bei Frage '24 [Bfremdspr]' (Beschäftigen Sie auf Ihrem Betrieb fremdsprachige Arbeitskräfte?)

❗ Bitte wählen Sie die zutreffenden Antworten aus:
Bitte wählen Sie alle zutreffenden Antworten aus:

- ☐ Melker
- ☐ Kälberversorgung
- ☐ Herdenmanagement
- ☐ Fütterung
- ☐ Ackerbau
- ☐ Allgemeine Stallhilfe
- ☐ Sonstiges: ____________

Gibt es gelegentlich sprachliche Verständigungsprobleme die negative Konsequenzen für ein Tier/ die Herde hatten? *

Beantworten Sie diese Frage nur, wenn folgende Bedingungen erfüllt sind:
Antwort war 'Ja' bei Frage '24 [Bfremdspr]' (Beschäftigen Sie auf Ihrem Betrieb fremdsprachige Arbeitskräfte?)

Bitte wählen Sie die zutreffende Antwort für jeden Punkt aus:

	**Nie**	**Selten**	**Gelegentlich**	**Oft**	**Immer**
	◯	◯	◯	◯	◯

Was beschreibt Ihr Verständnis von Integrierter Tierärztlicher Bestandsbetreuung? *

❶ Alle Ihre Antworten müssen unterschiedlich sein, und müssen zugeordnet sein.
Bitte nummerieren Sie jede Box in der Reihenfolge Ihrer Präferenz, beginnen mit 1 bis 5

[ ] Trächtigkeitsuntersuchungen/ Beratung zum Thema Reproduktion
[ ] Diskussion von Herdenproduktionsdaten
[ ] Bestandsrundgänge in den Produktionsabschnitten/ Strategiebesprechung
[ ] Erkennen und Beheben von aktuellen Bestandsproblemen
[ ] Verbesserung der Betriebswirtschaftlichkeit/ Kosten-Nutzen-Analyse

Nehmen Sie aktuell an ITB teil oder haben Sie in den letzten 5 Jahren teilgenommen? *

Bitte wählen Sie nur eine der folgenden Antworten aus:

- ◯ Ja
- ◯ Nein

Treffen Sie Entscheidungen für die Tiergesundheit in der Regel gemeinsam mit Ihrem Tierarzt? *

Bitte wählen Sie die zutreffende Antwort für jeden Punkt aus:

	Nie	Selten	Gelegentlich	Oft	Immer
	◯	◯	◯	◯	◯

Gesamtzufriedenheit

(Bitte bewerten Sie die Aussage anhand von Schulnoten mit 1 = sehr gut bis 5 = ungenügend.)

*

Bitte wählen Sie die zutreffende Antwort für jeden Punkt aus:

	1	2	3	4	5
**Mein Tierarzt betreut meinen Betrieb insgesamt zu meiner Zufriedenheit.**	◯	◯	◯	◯	◯

Haben Sie früher einmal an ITB teilgenommen? *

Beantworten Sie diese Frage nur, wenn folgende Bedingungen erfüllt sind:
Antwort war 'Nein' bei Frage '30 [CTeilnahme]' (Nehmen Sie aktuell an ITB teil oder haben Sie in den letzten 5 Jahren teilgenommen?)

Bitte wählen Sie nur eine der folgenden Antworten aus:

- ◯ Ja
- ◯ Nein

Was war Ihr Hauptgrund, die ITB zu kündigen? *

Beantworten Sie diese Frage nur, wenn folgende Bedingungen erfüllt sind:
Antwort war 'Ja' bei Frage '33 [CNfrueherteilgenomme]' (Haben Sie früher einmal an ITB teilgenommen?)

❶ Bitte wählen Sie eine der folgenden Antworten:
Bitte wählen Sie nur eine der folgenden Antworten aus:

- ◯ erfolglos
- ◯ zu teuer
- ◯ zu zeitintensiv
- ◯ Tierarztwechsel
- ◯ Sonstiges [ ]

Können Sie sich vorstellen, dass Ihr Betrieb Bedarf an ITB hat? *

Beantworten Sie diese Frage nur, wenn folgende Bedingungen erfüllt sind:
Antwort war 'Nein' bei Frage '30 [CTeilnahme]' (Nehmen Sie aktuell an ITB teil oder haben Sie in den letzten 5 Jahren teilgenommen?)

❗ Bitte wählen Sie eine der folgenden Antworten:
Bitte wählen Sie nur eine der folgenden Antworten aus:

- ◯ ja
- ◯ unsicher
- ◯ Nein, ITB ist für mich nicht nützlich, weil [ ]

Wenn Sie glauben, dass Ihr Betrieb kein Bedarf hat, tragen Sie bitte den Grund/ die Gründe in das freie Feld ein.

Wo könnte Ihr Betrieb Bedarf für ITB haben? *

Beantworten Sie diese Frage nur, wenn folgende Bedingungen erfüllt sind:
Antwort war 'unsicher' *oder* 'ja' bei Frage '35 [CNBedarf]' (Können Sie sich vorstellen, dass Ihr Betrieb Bedarf an ITB hat?)

❗ Bitte wählen Sie die zutreffenden Antworten aus:
Bitte wählen Sie alle zutreffenden Antworten aus:

- ☐ Fruchtbarkeit
- ☐ Eutergesundheit
- ☐ Leistung/ Auswertung von Herdendaten/ Digitalisierung im Kuhstall
- ☐ Klauengesundheit
- ☐ Jungtiergesundheit
- ☐ Tierernährung (Fütterungs- und Leistungsanalysen)
- ☐ Stallbau/ Tierhaltung
- ☐ Biosicherheit *
- ☐ Betriebswirtschaftlichkeit
- ☐ Tierwohl
- ☐ Angestelltenmanagement/ -ausbildung

* Biosicherheit beinhaltet alle Maßnahmen, um den Eintrag von gesundheitsrelevanten Mikroorganismen in einen Bestand (äußere Absicherung) und die Verschleppung innerhalb eines Betriebs (innere Absicherung) zu verhindern.

Wären Sie bereit, für tierärztliche Beratung den in der Gebührenordnung für Tierärzte festgelegten Mindeststundensatz von 89,32€ zu zahlen? *

Beantworten Sie diese Frage nur, wenn folgende Bedingungen erfüllt sind:
Antwort war 'Nein' bei Frage '30 [CTeilnahme]' (Nehmen Sie aktuell an ITB teil oder haben Sie in den letzten 5 Jahren teilgenommen?)

Bitte wählen Sie nur eine der folgenden Antworten aus:

- ◯ Ja
- ◯ Nein

Von wievielen Tierarztpraxen wird Ihr Betrieb betreut? *

❗ Bitte wählen Sie eine der folgenden Antworten:
Bitte wählen Sie nur eine der folgenden Antworten aus:

- ◯ 1
- ◯ 2
- ◯ 3 oder mehr

Welche Leistungen erbringen die verschiedenen Tierarztpraxen außerhalb der Ihres Hoftierarztes? *

Beantworten Sie diese Frage nur, wenn folgende Bedingungen erfüllt sind:
Antwort war '2' *oder* '3 oder mehr' bei Frage '38 [CNAnzahlTAP]' (Von wievielen Tierarztpraxen wird Ihr Betrieb betreut?)

❗ Bitte wählen Sie die zutreffenden Antworten aus:
Bitte wählen Sie alle zutreffenden Antworten aus:

- ☐ Trächtigkeitsuntersuchungen/ Fruchtbarkeits-Checks
- ☐ Medikamentenbezug
- ☐ Fütterungsberatung
- ☐ Sonstiges: [ ]

Sind in Ihrem Betrieb nichttierärztliche Berater tätig? *

Bitte wählen Sie nur eine der folgenden Antworten aus:

- ◯ Ja
- ◯ Nein

Nichttierärztliche Berater wie z.B. Fütterungsberater, Besamungstechniker, ...

Welche nichttierätztlichen Berater sind in Ihrem Betrieb tätig? *

Beantworten Sie diese Frage nur, wenn folgende Bedingungen erfüllt sind:
Antwort war 'Nein' bei Frage '30 [CTeilnahme]' (Nehmen Sie aktuell an ITB teil oder haben Sie in den letzten 5 Jahren teilgenommen?) *und* Antwort war 'Ja' bei Frage '40 [CNnichttierarztBer]' (Sind in Ihrem Betrieb nichttierärztliche Berater tätig?)

❶ Bitte wählen Sie die zutreffenden Antworten aus:
Bitte wählen Sie alle zutreffenden Antworten aus:

- ☐ Fütterungsberater
- ☐ Besamungstechniker/ Rinderzuchtverband
- ☐ Agrarberater
- ☐ Tiergesundheitsdienst
- ☐ Molkerei/ Abteilung Qualitätsmanagement
- ☐ Beratungsring
- ☐ Sonstiges: ____________

Wie lange nehmen Sie schon an ITB teil? *

Beantworten Sie diese Frage nur, wenn folgende Bedingungen erfüllt sind:
Antwort war 'Ja' bei Frage '30 [CTeilnahme]' (Nehmen Sie aktuell an ITB teil oder haben Sie in den letzten 5 Jahren teilgenommen?)

❶ Bitte wählen Sie eine der folgenden Antworten:
Bitte wählen Sie nur eine der folgenden Antworten aus:

- ◯ < 1 Jahr
- ◯ 1 - 5 Jahre
- ◯ > 5 Jahre

Wer führt die ITB auf Ihrem Betrieb durch? *

❶ Bitte wählen Sie eine der folgenden Antworten:
Bitte wählen Sie nur eine der folgenden Antworten aus:

- ◯ Hoftierarzt
- ◯ anderer, zusätzlicher Tierarzt
- ◯ Tiergesundheitsdienst (TGD)
- ◯ Universität
- ◯ Sonstiges ____________

Erfolgt der Besuch der ITB zu einem separaten Termin, unabhängig von Terminen für kranke Tiere? *

Beantworten Sie diese Frage nur, wenn folgende Bedingungen erfüllt sind:
((CTeilnahme.NAOK (/index.php/admin/questions/sa/view/surveyid/219865/gid/302/qid/7087) == "Y"))

Bitte wählen Sie nur eine der folgenden Antworten aus:

- ◯ Ja
- ◯ Nein

Wie oft werden reine Bestandsbetreuungsbesuche bei Ihnen durchgeführt? *

Beantworten Sie diese Frage nur, wenn folgende Bedingungen erfüllt sind:
Antwort war 'Ja' bei Frage '44 [DITBTermin]' (Erfolgt der Besuch der ITB zu einem separaten Termin, unabhängig von Terminen für kranke Tiere?)

❶ Bitte wählen Sie eine der folgenden Antworten:
Bitte wählen Sie nur eine der folgenden Antworten aus:

- ◯ öfter als 1x pro Monat
- ◯ 1x pro Monat
- ◯ 1x alle 2 Monate
- ◯ 1x alle 3 Monate
- ◯ seltener als alle 3 Monate

Wird in Ihrem Betrieb mindestens halbjährlich der aktuelle Zustand erhoben und auf dessen Grundlage die betrieblichen Ziele für die nächsten sechs Monate festgelegt? *

Beantworten Sie diese Frage nur, wenn folgende Bedingungen erfüllt sind:
((CTeilnahme.NAOK (/index.php/admin/questions/sa/view/surveyid/219865/gid/302/qid/7087) == "Y"))

Bitte wählen Sie nur eine der folgenden Antworten aus:

- ◯ Ja
- ◯ Nein

Haben Sie und Ihr Tierarzt gemeinsam schriftliche Ziele festgelegt, die Sie kurz-, mittel- und langfristig erreichen wollen? *

Beantworten Sie diese Frage nur, wenn folgende Bedingungen erfüllt sind:
((CTeilnahme.NAOK (/index.php/admin/questions/sa/view/surveyid/219865/gid/302/qid/7087) == "Y"))

Bitte wählen Sie nur eine der folgenden Antworten aus:

- ◯ Ja
- ◯ Nein

Wurde im Rahmen der ITB mindestens 1x eine Kosten-Nutzen-Analyse zu Investitionen in Tiergesundheit durch Ihren Tierarzt durchgeführt? *

Beantworten Sie diese Frage nur, wenn folgende Bedingungen erfüllt sind:
((CTeilnahme.NAOK (/index.php/admin/questions/sa/view/surveyid/219865/gid/302/qid/7087) == "Y"))

Bitte wählen Sie nur eine der folgenden Antworten aus:

- ◯ Ja
- ◯ Nein

Haben Sie schriftlich fixierte Arbeitsanweisungen für Gesundheitskontrollen, Vorsorgemaßnahmen und Behandlungen?

Gesundheitskontrolle (z.B. 10-Tage Temperaturmessen bei Frischabkalbern)

Vorsorgemaßnahme (z.B. Kälber innerhalb von 2h mit Kolostrum versorgen)

Behandlungen (z.B. erstes Vorgehen bei einer Kuh mit Verdacht auf Milchfieber)

*

Beantworten Sie diese Frage nur, wenn folgende Bedingungen erfüllt sind:
((CTeilnahme.NAOK (/index.php/admin/questions/sa/view/surveyid/219865/gid/302/qid/7087) == "Y"))

Bitte wählen Sie die zutreffende Antwort für jeden Punkt aus:

	Ja	Unsicher	Nein
	◯	◯	◯

Was ist Ihre Hauptmotivation an ITB teilzunehmen? *

Beantworten Sie diese Frage nur, wenn folgende Bedingungen erfüllt sind:
((CTeilnahme.NAOK (/index.php/admin/questions/sa/view/surveyid/219865/gid/302/qid/7087) == "Y"))

Bitte geben Sie Ihre Antwort hier ein:

Wie hoch ist Ihre jeweilige Motivation der einzelnen Aussagen an ITB teilzunehmen? *

Beantworten Sie diese Frage nur, wenn folgende Bedingungen erfüllt sind:
Antwort war größer als bei Frage '50 [DITBMotivationfrei]' (Was ist Ihre Hauptmotivation an ITB teilzunehmen?)

Bitte wählen Sie die zutreffende Antwort für jeden Punkt aus:

	trifft voll zu (++)	trifft zu (+)	neutral (0)	trifft nicht zu (-)	trifft gar nicht zu (--)
**Regelmäßige Kontrolle der Produktionsdaten**	◯	◯	◯	◯	◯
**Betriebsblindheit vorbeugen**	◯	◯	◯	◯	◯
**Teilnahme durch den Tierarzt empfohlen**	◯	◯	◯	◯	◯
**Höhere Gewinnerzielung**	◯	◯	◯	◯	◯
**Beheben eines Herdengesundheitsproblems**	◯	◯	◯	◯	◯
**Strukturiertere Arbeit/ Teilen von Verwaltungsarbeit mit dem Tierarzt**	◯	◯	◯	◯	◯
**Gesetzeskonflikte vermeiden (Arzneimittel, Tierschutz, ...)**	◯	◯	◯	◯	◯
**ITB-Teilnahme wurde mir vorgeschrieben (z.B. vom Veterinäramt, Molkerei, Schlachthof)**	◯	◯	◯	◯	◯

Bitte bewerten Sie die folgenden Aussagen.

Die Vorteile, die ich bisher durch ITB erfahren habe, sind *

Beantworten Sie diese Frage nur, wenn folgende Bedingungen erfüllt sind:
((CTeilnahme.NAOK (/index.php/admin/questions/sa/view/surveyid/219865/gid/302/qid/7087) == "Y"))

Bitte wählen Sie die zutreffende Antwort für jeden Punkt aus:

	trifft voll zu (++)	trifft zu (+)	neutral (0)	trifft nicht zu (-)	trifft gar nicht zu (--)
**Höhere Herdenleistung**	◯	◯	◯	◯	◯
**Besseres Betriebsmanagement**	◯	◯	◯	◯	◯
**Bessere Herdengesundheit (Auftreten/ Schwere von Krankheiten vermindert)**	◯	◯	◯	◯	◯
**Rechtzeitigere Erkennung von Problemen**	◯	◯	◯	◯	◯
**Regelmäßige Kontrolle von Produktionsdaten**	◯	◯	◯	◯	◯
**Verhinderung von Betriebsblindheit**	◯	◯	◯	◯	◯
**Zeitnahe Information über tiermedizinische Entwicklungen**	◯	◯	◯	◯	◯
**Strukturiertere Problemlösung**	◯	◯	◯	◯	◯

Die Nachteile, die ich bisher durch ITB erfahren habe, sind: *

Beantworten Sie diese Frage nur, wenn folgende Bedingungen erfüllt sind:
((CTeilnahme.NAOK (/index.php/admin/questions/sa/view/surveyid/219865/gid/302/qid/7087) == "Y"))

Bitte wählen Sie die zutreffende Antwort für jeden Punkt aus:

	trifft voll zu (++)	trifft zu (+)	neutral (0)	trifft nicht zu (-)	trifft gar nicht zu (--)
**Hohe Kosten**	◯	◯	◯	◯	◯
**Hoher Zeitaufwand**	◯	◯	◯	◯	◯
**Unpassende Besuchzeiten des Tierarztes**	◯	◯	◯	◯	◯
**Schwierigkeiten die nötigen Daten zu sammeln**	◯	◯	◯	◯	◯
**Tierarzt hat wenig Erfahrung/ gibt zu wenig gute Ratschläge**	◯	◯	◯	◯	◯
**Tierarzt mischt sich (zu viel) ins Management ein**	◯	◯	◯	◯	◯
**Ratschlag ist oft zu pauschal und nicht für meinen Betrieb passend**	◯	◯	◯	◯	◯
**Ich folge den Ratschlägen nicht, weil sie mir nicht sinnvoll erscheinen**	◯	◯	◯	◯	◯

Auswirkung der ITB auf die betriebliche Organisation *

Beantworten Sie diese Frage nur, wenn folgende Bedingungen erfüllt sind:
((CTeilnahme.NAOK (/index.php/admin/questions/sa/view/surveyid/219865/gid/302/qid/7087) == "Y"))

Bitte wählen Sie die zutreffende Antwort für jeden Punkt aus:

	trifft voll zu (++)	trifft zu (+)	neutral (0)	trifft nicht zu (-)	trifft gar nicht zu (--)
**Meine Organisation hat sich durch die Teilnahme an ITB verbessert.**	◯	◯	◯	◯	◯

Welche Bereiche werden in Ihrem Betrieb im Rahmen der ITB betreut? *

❶ Bitte wählen Sie die zutreffenden Antworten aus:
Bitte wählen Sie alle zutreffenden Antworten aus:

- [ ] Fruchtbarkeit
- [ ] Eutergesundheit
- [ ] Leistung/ Auswertung von Herdendaten/ Digitalisierung im Kuhstall
- [ ] Klauengesundheit
- [ ] Jungtiergesundheit
- [ ] Tierernährung (Fütterungs- und Leistungsanalysen)
- [ ] Stallbau/ Tierhaltung
- [ ] Biosicherheit
- [ ] Betriebswirtschaftlichkeit
- [ ] Tierwohl
- [ ] Angestelltenmanagement/ -ausbildung

* Biosicherheit beinhaltet alle Maßnahmen, um den Eintrag von gesundheitsrelevanten Mikroorganismen in einen Bestand (äußere Absicherung) und die Verschleppung innerhalb eines Betriebs (innere Absicherung) zu verhindern.

Fruchtbarkeit

Erfüllt Ihr Tierarzt Ihre Erwartungen hinsichtlich des Bereichs "Fruchtbarkeit"?

(Bitte bewerten Sie die Aussage anhand von Schulnoten mit 1 = sehr gut bis 5 = ungenügend.)

*

Beantworten Sie diese Frage nur, wenn folgende Bedingungen erfüllt sind:
Antwort war 'Fruchtbarkeit' bei Frage '55 [EITBBereiche]' (Welche Bereiche werden in Ihrem Betrieb im Rahmen der ITB betreut?)

Bitte wählen Sie die zutreffende Antwort für jeden Punkt aus:

	1	2	3	4	5
**Fruchtbarkeit**	○	○	○	○	○

Eutergesundheit

Erfüllt Ihr Tierarzt Ihre Erwartungen hinsichtlich des Bereichs "Eutergesundheit"?

(Bitte bewerten Sie die Aussage anhand von Schulnoten mit 1 = sehr gut bis 5 = ungenügend.)

*

Beantworten Sie diese Frage nur, wenn folgende Bedingungen erfüllt sind:
Antwort war 'Eutergesundheit' bei Frage '55 [EITBBereiche]' (Welche Bereiche werden in Ihrem Betrieb im Rahmen der ITB betreut?)

Bitte wählen Sie die zutreffende Antwort für jeden Punkt aus:

	1	2	3	4	5
**Eutergesundheit**	○	○	○	○	○

Leistung/ Auswertung von Herdendaten/ Digitalisierung im Kuhstall

Erfüllt Ihr Tierarzt Ihre Erwartungen hinsichtlich des Bereichs "Leistung/ Auswertung von Herdendaten/ Digitalisierung im Kuhstall"?

(Bitte bewerten Sie die Aussage anhand von Schulnoten mit 1 = sehr gut bis 5 = ungenügend.)

*

Beantworten Sie diese Frage nur, wenn folgende Bedingungen erfüllt sind:
Antwort war 'Leistung/ Auswertung von Herdendaten/ Digitalisierung im Kuhstall' bei Frage '55 [EITBBereiche]' (Welche Bereiche werden in Ihrem Betrieb im Rahmen der ITB betreut?)

Bitte wählen Sie die zutreffende Antwort für jeden Punkt aus:

	1	2	3	4	5
**Leistung/ Auswertung von Herdendaten/ Digitalisierung im Kuhstall**	○	○	○	○	○

Klauengesundheit

Erfüllt Ihr Tierarzt Ihre Erwartungen hinsichtlich des Bereichs "Klauengesundheit"?

(Bitte bewerten Sie die Aussage anhand von Schulnoten mit 1 = sehr gut bis 5 = ungenügend.)

*

Beantworten Sie diese Frage nur, wenn folgende Bedingungen erfüllt sind:
Antwort war 'Klauengesundheit' bei Frage '55 [EITBBereiche]' (Welche Bereiche werden in Ihrem Betrieb im Rahmen der ITB betreut?)

Bitte wählen Sie die zutreffende Antwort für jeden Punkt aus:

	1	2	3	4	5
**Klauengesundheit**	◯	◯	◯	◯	◯

Jungtiergesundheit

Erfüllt Ihr Tierarzt Ihre Erwartungen hinsichtlich des Bereichs "Jungtiergesundheit"?

(Bitte bewerten Sie die Aussage anhand von Schulnoten mit 1 = sehr gut bis 5 = ungenügend.)

*

Beantworten Sie diese Frage nur, wenn folgende Bedingungen erfüllt sind:
Antwort war 'Jungtiergesundheit' bei Frage '55 [EITBBereiche]' (Welche Bereiche werden in Ihrem Betrieb im Rahmen der ITB betreut?)

Bitte wählen Sie die zutreffende Antwort für jeden Punkt aus:

	1	2	3	4	5
**Jungtiergesundheit**	◯	◯	◯	◯	◯

Tierernährung (Fütterungs- und Leistungsanalysen)

Erfüllt Ihr Tierarzt Ihre Erwartungen hinsichtlich des Bereichs "Tierernährung (Fütterungs- und Leistungsanalysen)"?

(Bitte bewerten Sie die Aussage anhand von Schulnoten mit 1 = sehr gut bis 5 = ungenügend.)

*

Beantworten Sie diese Frage nur, wenn folgende Bedingungen erfüllt sind:
Antwort war 'Tierernährung (Fütterungs- und Leistungsanalysen)' bei Frage '55 [EITBBereiche]' (Welche Bereiche werden in Ihrem Betrieb im Rahmen der ITB betreut?)

Bitte wählen Sie die zutreffende Antwort für jeden Punkt aus:

	1	2	3	4	5
**Tierernährung (Fütterungs- und Leistungsanalysen)**	◯	◯	◯	◯	◯

Stallbau/ Tierhaltung

Erfüllt Ihr Tierarzt Ihre Erwartungen hinsichtlich des Bereichs "Stallbau/ Tierhaltung"?

(Bitte bewerten Sie die Aussage anhand von Schulnoten mit 1 = sehr gut bis 5 = ungenügend.)

*

Beantworten Sie diese Frage nur, wenn folgende Bedingungen erfüllt sind:
Antwort war 'Stallbau/ Tierhaltung' bei Frage '55 [EITBBereiche]' (Welche Bereiche werden in Ihrem Betrieb im Rahmen der ITB betreut?)

Bitte wählen Sie die zutreffende Antwort für jeden Punkt aus:

	1	2	3	4	5
**Stallbau/ Tierhaltung**	◯	◯	◯	◯	◯

Biosicherheit

Erfüllt Ihr Tierarzt Ihre Erwartungen hinsichtlich des Bereichs "Biosicherheit"?

(Bitte bewerten Sie die Aussage anhand von Schulnoten mit 1 = sehr gut bis 5 = ungenügend.)

*

Beantworten Sie diese Frage nur, wenn folgende Bedingungen erfüllt sind:
Antwort war 'Biosicherheit' bei Frage '55 [EITBBereiche]' (Welche Bereiche werden in Ihrem Betrieb im Rahmen der ITB betreut?)

Bitte wählen Sie die zutreffende Antwort für jeden Punkt aus:

	1	2	3	4	5
**Biosicherheit**	◯	◯	◯	◯	◯

Betriebswirtschaftlichkeit

Erfüllt Ihr Tierarzt Ihre Erwartungen hinsichtlich des Bereichs "Betriebswirtschaftlichkeit"?

(Bitte bewerten Sie die Aussage anhand von Schulnoten mit 1 = sehr gut bis 5 = ungenügend.)

*

Beantworten Sie diese Frage nur, wenn folgende Bedingungen erfüllt sind:
Antwort war 'Betriebswirtschaftlichkeit' bei Frage '55 [EITBBereiche]' (Welche Bereiche werden in Ihrem Betrieb im Rahmen der ITB betreut?)

Bitte wählen Sie die zutreffende Antwort für jeden Punkt aus:

	1	2	3	4	5
**Betriebswirtschaftlichkeit**	◯	◯	◯	◯	◯

Tierwohl

Erfüllt Ihr Tierarzt Ihre Erwartungen hinsichtlich des Bereichs "Tierwohl"?

(Bitte bewerten Sie die Aussage anhand von Schulnoten mit 1 = sehr gut bis 5 = ungenügend.)

*

Beantworten Sie diese Frage nur, wenn folgende Bedingungen erfüllt sind:
Antwort war 'Tierwohl' bei Frage '55 [EITBBereiche]' (Welche Bereiche werden in Ihrem Betrieb im Rahmen der ITB betreut?)

Bitte wählen Sie die zutreffende Antwort für jeden Punkt aus:

	1	2	3	4	5
**Tierwohl**	◯	◯	◯	◯	◯

Angestelltenmanagement/ -ausbildung

Erfüllt Ihr Tierarzt Ihre Erwartungen hinsichtlich des Bereichs "Angestelltenmanagement/ -ausbildung"?

(Bitte bewerten Sie die Aussage anhand von Schulnoten mit 1 = sehr gut bis 5 = ungenügend.)

*

Beantworten Sie diese Frage nur, wenn folgende Bedingungen erfüllt sind:
Antwort war 'Angestelltenmanagement/ -ausbildung' bei Frage '55 [EITBBereiche]' (Welche Bereiche werden in Ihrem Betrieb im Rahmen der ITB betreut?)

Bitte wählen Sie die zutreffende Antwort für jeden Punkt aus:

	1	2	3	4	5
**Angestelltenmanagement/ -ausbildung**	◯	◯	◯	◯	◯

Wie bewerten Sie die Zusammenarbeit mit Ihrem Tierarzt im Rahmen der ITB? *

Bitte wählen Sie die zutreffende Antwort für jeden Punkt aus:

	trifft voll zu (++)	trifft zu (+)	neutral (0)	trifft nicht zu (-)	trifft gar nicht zu (--)
**Mein Tierarzt bereitet sich auf den Bestandsbesuch vor.**	◯	◯	◯	◯	◯
**Mein Tierarzt knüpft an Themen des vorherigen Bestandsbesuches an.**	◯	◯	◯	◯	◯
**Mein Tierarzt erläutert mir Vor- und Nachteile von möglichen Maßnahmen.**	◯	◯	◯	◯	◯
**Die Ratschläge sind gut praktisch umzusetzen.**	◯	◯	◯	◯	◯
**Mein Tierarzt trifft mit mir eindeutige Vereinbarungen, wenn Maßnahmen keine Wirkung zeigen.**	◯	◯	◯	◯	◯
**Mein Tierarzt hält sich immer an Absprachen.**	◯	◯	◯	◯	◯
**Mein Tierarzt ist immer pünktlich.**	◯	◯	◯	◯	◯
**Mein Tierarzt hört mir aufmerksam zu und hat genug Zeit für meine Fragen.**	◯	◯	◯	◯	◯
**Mein Tierarzt verwendet für seine Erklärungen eine präzise und leicht verständliche Sprache.**	◯	◯	◯	◯	◯
**Mein Tierarzt erklärt mir Ergebnisse von Untersuchungen (z.B. Laborergebnisse) genau.**	◯	◯	◯	◯	◯
**Ich würde mir von meinem Tierarzt mehr fachliche Information/ Empfehlung wünschen.**	◯	◯	◯	◯	◯
**Der Fortschritt durch die ITB zahlt den Aufwand/ Kosten aus.**	◯	◯	◯	◯	◯

Was bietet Ihr Tierarzt als Teil der bestandsbetreuenden Tätigkeit für Leistungen an? *

❶ Bitte wählen Sie die zutreffenden Antworten aus:
Bitte wählen Sie alle zutreffenden Antworten aus:

- ☐ keine
- ☐ Laboruntersuchungen *
- ☐ regelmäßiger Fachinformationsbrief der Praxis
- ☐ Informations-/ Themenveranstaltungen mit anderen Landwirten der Praxis
- ☐ Schulungen auf Betrieben zum Tierumgang und Erkennung von Krankheiten mit anderen Landwirten der Praxis
- ☐ Futtermittelanalysen
- ☐ Sonstiges: ______

* z.B. Blutproben, Milchproben, Harnproben, Kotproben als Teil der Bestandsbetreuung; z.B. zur Identifikation eines Herdenproblems

Wenn eine tierärztliche Empfehlung nicht das gewünschte Resultat hat, was ist oft der Grund? *

❶ Bitte wählen Sie eine der folgenden Antworten:
Bitte wählen Sie nur eine der folgenden Antworten aus:

- ◯ Ich bin der Empfehlung gefolgt, aber es scheiterte, weil die Empfehlung nicht die Ursache des Problems korrekt behoben hat.
- ◯ Ich bin der Empfehlung gefolgt, aber es scheiterte, weil die korrekte Ursache adressiert und auch umgesetzt wurde, aber dennoch kein Effekt auftrat.
- ◯ Ich bin der Empfehlung NICHT gefolgt, weil die Empfehlung nicht praktikabel und im Alltag umsetzbar war.
- ◯ Ich bin der Empfehlung NICHT gefolgt, weil die Empfehlung mir nicht nützlich schien.
- ◯ Ich bin der Empfehlung NICHT gefolgt, weil die Empfehlung schwierig zu verstehen war.

Wie wird für ITB auf Ihrem Betrieb abgerechnet?

(Bitte tragen Sie in das Kommentarfeld die zutreffende Gebühr für Ihre Abrechnung ein.)

*

❶ Bitte wählen Sie eine der folgenden Antworten:
Bitte wählen Sie nur eine der folgenden Antworten aus:

- ◯ Stundensatz von ___ € (inkl. aller durchgeführten beratenden und praktischen Leistungen)
- ◯ Stundensatz von ___ € (durchgeführte praktische Leistungen werden extra berechnet)
- ◯ Fester Satz pro Tier und Jahr von ___ €/ Tier/ Jahr
- ◯ Modul-Form: z.B. Fruchtbarkeits-Modul, in dem die Versorgung und Beratung rund um dieses Thema enthalten ist (Euter-Modul, Klauen-Modul, …): ___ €/ Modul
- ◯ Gesamtpaket/ Flatrate für den Bestand mit einer monatlichen Rate von ___ €
- ◯ Über ein (monatliches) Erfolgshonorar von ___ %

Bitte schreiben Sie einen Kommentar zu Ihrer Auswahl

Wie würden Sie sich die Abrechnung für ITB wünschen? *

❶ Bitte wählen Sie eine der folgenden Antworten:
Bitte wählen Sie nur eine der folgenden Antworten aus:

- ◯ Stundensatz (inkl. aller durchgeführten beratenden und praktischen Leistungen)
- ◯ Stundensatz (durchgeführte praktische Leistungen werden extra berechnet)
- ◯ Fester Satz pro Tier und Jahr
- ◯ Modul-Form
- ◯ Gesamtpaket/ Flatrate für den Bestand
- ◯ Erfolgshonorar

Erhalten Sie eine separate Rechnung für die Besuche im Rahmen der ITB? *

❶ Bitte wählen Sie eine der folgenden Antworten:
Bitte wählen Sie nur eine der folgenden Antworten aus:

- ◯ Ja, sie stehen gesondert und sind gut zu differenzieren.
- ◯ Nein, es gibt eine Rechnung für alles.

Wird die Vor- und Nachbereitungszeit des Tierarztes (abseits von Ihrem Betrieb) abgerechnet? *

❶ Bitte wählen Sie eine der folgenden Antworten:
Bitte wählen Sie nur eine der folgenden Antworten aus:

- ◯ Nein, wird nicht abgerechnet.
- ◯ Ja, wird abgerechnet, aber nicht separat.
- ◯ Ja, wird separat abgerechnet.
- ◯ Weiß nicht.

Glauben Sie, durch die ITB einen finanziellen Mehrwert in Ihrem Betrieb zu bekommen? *

Bitte wählen Sie die zutreffende Antwort für jeden Punkt aus:

	**trifft voll zu (++)**	**trifft zu (+)**	**neutral (0)**	**trifft nicht zu (-)**	**trifft gar nicht zu (--)**
**Glauben Sie, durch die ITB einen finanziellen Mehrwert in Ihrem Betrieb zu bekommen?**	◯	◯	◯	◯	◯

Wenn Ihre jetzige Gebühr für ITB um 10% steigen würde, würden Sie weiterhin teilnehmen? *

❶ Bitte wählen Sie eine der folgenden Antworten:
Bitte wählen Sie nur eine der folgenden Antworten aus:

- ◯ Ja, mit der gleichen Stundenanzahl.
- ◯ Ja, aber ich würde die Stundenanzahl reduzieren.
- ◯ Nein, ich würde dann aufhören daran teilzunehmen.

Unten stehen Aussagen zur Qualität der jetzigen ITB auf Ihrem Betrieb.

(Bitte bewerten Sie die Aussage anhand von Schulnoten mit 1 = sehr gut bis 5 = ungenügend.)

*

Bitte wählen Sie die zutreffende Antwort für jeden Punkt aus:

	1	2	3	4	5
**Themen und Inhalt der Bestandsbetreuung.**	◯	◯	◯	◯	◯
**Struktur und Ablauf der Bestandsbetreuung.**	◯	◯	◯	◯	◯
**Berücksichtigung und Erreichung MEINER Ziele und Prioritäten.**	◯	◯	◯	◯	◯
**Die Hintergründe der Ratschläge sind ausreichend erklärt und machen für mich Sinn.**	◯	◯	◯	◯	◯
**Absprache zwischen Tierarzt und anderen Beratern.**	◯	◯	◯	◯	◯
**Einsatz von Kosten-Nutzen-Analyse, um Entscheidungen zu treffen.**	◯	◯	◯	◯	◯
**Häufigkeit an Besuchen (Besuchsturnus).**	◯	◯	◯	◯	◯

Gibt es Themen, die Sie im Rahmen der ITB aufnehmen oder intensiver bearbeiten möchten?

(Bitte tragen Sie Ihre Wunschthemen in das nebenstehende Feld ein.)

*

❶ Bitte wählen Sie eine der folgenden Antworten:
❶ Wenn Sie 'Ja, z.B. folgende:' auswählen, spezifizieren Sie bitte Ihre Auswahl im entsprechenden Textfeld.
Bitte wählen Sie nur eine der folgenden Antworten aus:

- ◯ Nein
- ◯ Weiß nicht
- ◯ Ja, z.B. folgende: ____________

Fruchtbarkeit

Werden Sie zu diesem Thema von Ihrem Tierarzt betreut?

*

Bitte wählen Sie die zutreffende Antwort für jeden Punkt aus:

	Ja	Unsicher	Nein
	◯	◯	◯

Fruchtbarkeit

Was beinhaltet bei Ihnen Bestandsbetreuung auf diesem Gebiet bzw. in welchem Ausmaß gibt es Beratung?

*

Beantworten Sie diese Frage nur, wenn folgende Bedingungen erfüllt sind:
Antwort war 'Ja' *oder* 'Unsicher' bei Frage '78 [HFruchtbarkeit]' (Fruchtbarkeit Werden Sie zu diesem Thema von Ihrem Tierarzt betreut? ())

Bitte wählen Sie die zutreffende Antwort für jeden Punkt aus:

	Ja	Unsicher	Nein
**Trächtigkeitsuntersuchungen**	◯	◯	◯
**Puerperalkontrollen (nach der Kalbung)/ Sterilitätskontrolle**	◯	◯	◯
**Anpaarungsempfehlung**	◯	◯	◯
**Einsatz von Hormonprogrammen**	◯	◯	◯

Fruchtbarkeit

Warum ist diese Rubrik nicht Teil Ihrer Bestandsbetreuung?

*

Beantworten Sie diese Frage nur, wenn folgende Bedingungen erfüllt sind:
Antwort war 'Nein' bei Frage '78 [HFruchtbarkeit]' (Fruchtbarkeit Werden Sie zu diesem Thema von Ihrem Tierarzt betreut? ())

❗ Bitte wählen Sie eine der folgenden Antworten:
❗ Wenn Sie 'Sonstiges:' auswählen, spezifizieren Sie bitte Ihre Auswahl im entsprechenden Textfeld.
Bitte wählen Sie nur eine der folgenden Antworten aus:

◯ Der Besamungstechniker/ Rinderzuchtverband führt dies durch.
◯ Das kostet mir zu viel Zeit.
◯ Ich habe daran kein Interesse/ keine Probleme.
◯ Ich bezweifle, dass der Tierarzt über ausreichend Wissen und Expertise verfügt
◯ Es ist zu teuer, wenn der Tierarzt das macht.
◯ Mein Tierarzt hat es noch nie angeboten bzw. ich wusste nicht, dass das möglich ist.
◯ Sonstiges ____________

Fruchtbarkeit

Was würden Sie sich wünschen bzw. was fehlt Ihnen für eine gute Bestandsbetreuung?

Beantworten Sie diese Frage nur, wenn folgende Bedingungen erfüllt sind:
Antwort war 'Ja' *oder* 'Unsicher' *oder* 'Nein' bei Frage '78 [HFruchtbarkeit]' (Fruchtbarkeit Werden Sie zu diesem Thema von Ihrem Tierarzt betreut? ())

Bitte geben Sie Ihre Antwort hier ein:

____

Eutergesundheit/ Milchleistung/ -qualität/ Datenauswertung

Werden Sie zu diesem Thema von Ihrem Tierarzt betreut?

*

Bitte wählen Sie die zutreffende Antwort für jeden Punkt aus:

	Ja	Unsicher	Nein
	◯	◯	◯

Eutergesundheit/ Milchleistung/ -qualität/ Datenauswertung

Was beinhaltet bei Ihnen Bestandsbetreuung auf diesem Gebiet bzw. in welchem Ausmaß gibt es Beratung?

*

Beantworten Sie diese Frage nur, wenn folgende Bedingungen erfüllt sind:
Antwort war 'Ja' *oder* 'Unsicher' bei Frage '82 [HEuter]' (Eutergesundheit/ Milchleistung/ -qualität/ Datenauswertung Werden Sie zu diesem Thema von Ihrem Tierarzt betreut? ())

Bitte wählen Sie die zutreffende Antwort für jeden Punkt aus:

	Ja	Unsicher	Nein
**Auswertung der Milchleistungsprüfung/ anderer Daten**	○	○	○
**Milchproben nehmen, z.B. zur Leitkeimbestimmung**	○	○	○
**Überwachung des Melkprozess/ Melkroutine (z.B. Zitzenscoring)**	○	○	○

Eutergesundheit/ Milchleistung/ -qualität/ Datenauswertung

Warum ist diese Rubrik nicht Teil Ihrer Bestandsbetreuung?

*

Beantworten Sie diese Frage nur, wenn folgende Bedingungen erfüllt sind:
Antwort war 'Nein' bei Frage '82 [HEuter]' (Eutergesundheit/ Milchleistung/ -qualität/ Datenauswertung Werden Sie zu diesem Thema von Ihrem Tierarzt betreut? ())

❗ Bitte wählen Sie eine der folgenden Antworten:
❗ Wenn Sie 'Sonstiges:' auswählen, spezifizieren Sie bitte Ihre Auswahl im entsprechenden Textfeld.
Bitte wählen Sie nur eine der folgenden Antworten aus:

- ○ Ein anderer Berater führt dies durch.
- ○ Das kostet mir zu viel Zeit.
- ○ Ich habe daran kein Interesse/ keine Probleme.
- ○ Ich bezweifle, dass der Tierarzt über ausreichend Wissen und Expertise verfügt
- ○ Es ist zu teuer, wenn der Tierarzt das macht.
- ○ Mein Tierarzt hat es noch nie angeboten bzw. ich wusste nicht, dass das möglich ist.
- ○ Sonstiges ____________

Eutergesundheit/ Milchleistung/ -qualität/ Datenauswertung

Was würden Sie sich wünschen bzw. was fehlt Ihnen für eine gute Bestandsbetreuung?

Beantworten Sie diese Frage nur, wenn folgende Bedingungen erfüllt sind:
Antwort war 'Ja' *oder* 'Unsicher' *oder* 'Nein' bei Frage '82 [HEuter]' (Eutergesundheit/ Milchleistung/ -qualität/ Datenauswertung Werden Sie zu diesem Thema von Ihrem Tierarzt betreut? ())

Bitte geben Sie Ihre Antwort hier ein:

____

Klauengesundheit

Werden Sie zu diesem Thema von Ihrem Tierarzt betreut?

*

Bitte wählen Sie die zutreffende Antwort für jeden Punkt aus:

	Ja	Unsicher	Nein
	○	○	○

Klauengesundheit

Was beinhaltet bei Ihnen Bestandsbetreuung auf diesem Gebiet bzw. in welchem Ausmaß gibt es Beratung?

*

Beantworten Sie diese Frage nur, wenn folgende Bedingungen erfüllt sind:
Antwort war 'Ja' *oder* 'Unsicher' bei Frage '86 [HKlauen]' (Klauengesundheit Werden Sie zu diesem Thema von Ihrem Tierarzt betreut? ())

Bitte wählen Sie die zutreffende Antwort für jeden Punkt aus:

	Ja	Unsicher	Nein
**Praktische Klauenpflege/ Herdenschnitt**	◯	◯	◯
**regelmäßige Lahmheitsüberwachung ("Locomotion Scoring")**	◯	◯	◯
**Auswertung von Klauenschnittdaten (Krankheiten)**	◯	◯	◯

Klauengesundheit

Warum ist diese Rubrik nicht Teil Ihrer Bestandsbetreuung?

*

Beantworten Sie diese Frage nur, wenn folgende Bedingungen erfüllt sind:
Antwort war 'Nein' bei Frage '86 [HKlauen]' (Klauengesundheit Werden Sie zu diesem Thema von Ihrem Tierarzt betreut? ())

❗ Bitte wählen Sie eine der folgenden Antworten:
❗ Wenn Sie 'Sonstiges:' auswählen, spezifizieren Sie bitte Ihre Auswahl im entsprechenden Textfeld.
Bitte wählen Sie nur eine der folgenden Antworten aus:

◯ Der Klauenschneider führt dies durch.
◯ Das kostet mir zu viel Zeit.
◯ Ich habe daran kein Interesse/ keine Probleme.
◯ Ich bezweifle, dass der Tierarzt über ausreichend Wissen und Expertise verfügt
◯ Es ist zu teuer, wenn der Tierarzt das macht.
◯ Mein Tierarzt hat es noch nie angeboten bzw. ich wusste nicht, dass das möglich ist.
◯ Sonstiges

Klauengesundheit

Was würden Sie sich noch wünschen bzw. was fehlt Ihnen für eine gute Bestandsbetreuung?

Beantworten Sie diese Frage nur, wenn folgende Bedingungen erfüllt sind:
Antwort war 'Ja' *oder* 'Unsicher' *oder* 'Nein' bei Frage '86 [HKlauen]' (Klauengesundheit Werden Sie zu diesem Thema von Ihrem Tierarzt betreut? ())

Bitte geben Sie Ihre Antwort hier ein:

Jungtiergesundheit

Werden Sie zu diesem Thema von Ihrem Tierarzt betreut?

*

Bitte wählen Sie die zutreffende Antwort für jeden Punkt aus:

	Ja	Unsicher	Nein
	◯	◯	◯

Jungtiergesundheit

Was beinhaltet bei Ihnen Bestandsbetreuung auf diesem Gebiet bzw. in welchem Ausmaß gibt es Beratung?

*

Beantworten Sie diese Frage nur, wenn folgende Bedingungen erfüllt sind:
Antwort war 'Ja' *oder* 'Unsicher' bei Frage '90 [HJungtier]' (Jungtiergesundheit Werden Sie zu diesem Thema von Ihrem Tierarzt betreut? ())

Bitte wählen Sie die zutreffende Antwort für jeden Punkt aus:

	Ja	Unsicher	Nein
**Beratung zu Impfung und Entwurmung**	◯	◯	◯
**Beratung zur Fütterung (z.B. Tränkeplan etc.)**	◯	◯	◯
**Beratung zur Aufstallung (z.B. Vor-/Nachteile Einzel-/ Gruppenhaltung)**	◯	◯	◯
**Überwachung der Kolostrumversorgung durch Blutprobennahme**	◯	◯	◯

Jungtiergesundheit

Warum ist diese Rubrik nicht Teil Ihrer Bestandsbetreuung?

*

Beantworten Sie diese Frage nur, wenn folgende Bedingungen erfüllt sind:
Antwort war 'Nein' bei Frage '90 [HJungtier]' (Jungtiergesundheit Werden Sie zu diesem Thema von Ihrem Tierarzt betreut? ())

❗ Bitte wählen Sie eine der folgenden Antworten:
❗ Wenn Sie 'Sonstiges:' auswählen, spezifizieren Sie bitte Ihre Auswahl im entsprechenden Textfeld.
Bitte wählen Sie nur eine der folgenden Antworten aus:

- ◯ Ein anderer Berater führt dies durch.
- ◯ Das kostet mir zu viel Zeit.
- ◯ Ich habe daran kein Interesse/ keine Probleme.
- ◯ Ich bezweifle, dass der Tierarzt über ausreichend Wissen und Expertise verfügt
- ◯ Es ist zu teuer, wenn der Tierarzt das macht.
- ◯ Mein Tierarzt hat es noch nie angeboten bzw. ich wusste nicht, dass das möglich ist.
- ◯ Sonstiges ____________

Jungtiergesundheit

Was würden Sie sich wünschen bzw. was fehlt Ihnen für eine gute Bestandsbetreuung?

Beantworten Sie diese Frage nur, wenn folgende Bedingungen erfüllt sind:
Antwort war 'Ja' *oder* 'Unsicher' *oder* 'Nein' bei Frage '90 [HJungtier]' (Jungtiergesundheit Werden Sie zu diesem Thema von Ihrem Tierarzt betreut? ())

Bitte geben Sie Ihre Antwort hier ein:

____

Tierernährung (Fütterungs- und Leistungsanalysen)

Werden Sie zu diesem Thema von Ihrem Tierarzt betreut?

*

Bitte wählen Sie die zutreffende Antwort für jeden Punkt aus:

	Ja	Unsicher	Nein
	◯	◯	◯

Tierernährung (Fütterungs- und Leistungsanalysen)

Was beinhaltet bei Ihnen Bestandsbetreuung auf diesem Gebiet bzw. in welchem Ausmaß gibt es Beratung?

*

Beantworten Sie diese Frage nur, wenn folgende Bedingungen erfüllt sind:
Antwort war 'Ja' *oder* 'Unsicher' bei Frage '94 [HTierernaehrung]' (Tierernährung (Fütterungs- und Leistungsanalysen) Werden Sie zu diesem Thema von Ihrem Tierarzt betreut? ())

Bitte wählen Sie die zutreffende Antwort für jeden Punkt aus:

	Ja	Unsicher	Nein
**Beratung in Bezug auf MLP Ergebnisse/ Milchleistung**	◯	◯	◯
**Rationsberechnung**	◯	◯	◯
**Planung des Futtermittelanbaus**	◯	◯	◯
**Durchführung von TMR-Audits ***	◯	◯	◯
**Überwachung des Fütterungsmanagements (z.B. durch Zeitrafferkamera)**	◯	◯	◯

* TMR Audit: Betriebsspezifische Auswertung von Futterlagerung/ -vorbereitung, Mischvorgang, TMR-Vorlage sowie deren Arbeits- und Energieaufwand

Tierernährung (Fütterungs- und Leistungsanalysen

Warum ist diese Rubrik nicht Teil Ihrer Bestandsbetreuung?

*

Beantworten Sie diese Frage nur, wenn folgende Bedingungen erfüllt sind:
Antwort war 'Nein' bei Frage '94 [HTierernaehrung]' (Tierernährung (Fütterungs- und Leistungsanalysen) Werden Sie zu diesem Thema von Ihrem Tierarzt betreut? ())

❗ Bitte wählen Sie eine der folgenden Antworten:
❗ Wenn Sie 'Sonstiges:' auswählen, spezifizieren Sie bitte Ihre Auswahl im entsprechenden Textfeld.
Bitte wählen Sie nur eine der folgenden Antworten aus:

◯ Der Fütterungsberater führt dies durch.
◯ Das kostet mir zu viel Zeit.
◯ Ich habe daran kein Interesse/ keine Probleme.
◯ Ich bezweifle, dass der Tierarzt über ausreichend Wissen und Expertise verfügt
◯ Es ist zu teuer, wenn der Tierarzt das macht.
◯ Mein Tierarzt hat es noch nie angeboten bzw. ich wusste nicht, dass das möglich ist.
◯ Sonstiges

Tierernährung (Fütterungs- und Leistungsanalysen)

Was würden Sie sich wünschen bzw. was fehlt Ihnen für eine gute Bestandsbetreuung?

Beantworten Sie diese Frage nur, wenn folgende Bedingungen erfüllt sind:
Antwort war 'Ja' *oder* 'Unsicher' *oder* 'Nein' bei Frage '94 [HTierernaehrung]' (Tierernährung (Fütterungs- und Leistungsanalysen) Werden Sie zu diesem Thema von Ihrem Tierarzt betreut? ())

Bitte geben Sie Ihre Antwort hier ein:

Stallbau

Werden Sie zu diesem Thema von Ihrem Tierarzt betreut?

*

Bitte wählen Sie die zutreffende Antwort für jeden Punkt aus:

	Ja	Unsicher	Nein
	◯	◯	◯

Stallbau

Was beinhaltet bei Ihnen Bestandsbetreuung auf diesem Gebiet bzw. in welchem Ausmaß gibt es Beratung?

*

Beantworten Sie diese Frage nur, wenn folgende Bedingungen erfüllt sind:
Antwort war 'Ja' *oder* 'Unsicher' bei Frage '98 [HStallbau]' (Stallbau Werden Sie zu diesem Thema von Ihrem Tierarzt betreut? ())

Bitte wählen Sie die zutreffende Antwort für jeden Punkt aus:

	Ja	Unsicher	Nein
**Beratung zu baulichen Veränderungen (z.B. Bodenbelag, Boxenbeschaffenheit, ...)**	◯	◯	◯
**Beratung zu Stallklima/ Belüftung**	◯	◯	◯
**Klimamessung (inkl. Rauchtest)**	◯	◯	◯
**Beratung bei Stallneubau**	◯	◯	◯

Stallbau/ Tierhaltung

Warum ist diese Rubrik nicht Teil Ihrer Bestandsbetreuung?

*

Beantworten Sie diese Frage nur, wenn folgende Bedingungen erfüllt sind:
Antwort war 'Nein' bei Frage '98 [HStallbau]' (Stallbau Werden Sie zu diesem Thema von Ihrem Tierarzt betreut? ())

❗ Bitte wählen Sie eine der folgenden Antworten:
❗ Wenn Sie 'Sonstiges:' auswählen, spezifizieren Sie bitte Ihre Auswahl im entsprechenden Textfeld.
Bitte wählen Sie nur eine der folgenden Antworten aus:

- ◯ Ein anderer Berater führt dies durch.
- ◯ Das kostet mir zu viel Zeit.
- ◯ Ich habe daran kein Interesse/ keine Probleme.
- ◯ Ich bezweifle, dass der Tierarzt über ausreichend Wissen und Expertise verfügt
- ◯ Es ist zu teuer, wenn der Tierarzt das macht.
- ◯ Mein Tierarzt hat es noch nie angeboten bzw. ich wusste nicht, dass das möglich ist.
- ◯ Sonstiges ____________

Stallbau

Was würden Sie sich wünschen bzw. was fehlt Ihnen für eine gute Bestandsbetreuung?

Beantworten Sie diese Frage nur, wenn folgende Bedingungen erfüllt sind:
Antwort war 'Ja' *oder* 'Unsicher' *oder* 'Nein' bei Frage '98 [HStallbau]' (Stallbau Werden Sie zu diesem Thema von Ihrem Tierarzt betreut? ())

Bitte geben Sie Ihre Antwort hier ein:

____

Biosicherheit

Werden Sie zu diesem Thema von Ihrem Tierarzt betreut?

*

Bitte wählen Sie die zutreffende Antwort für jeden Punkt aus:

	Ja	Unsicher	Nein
	◯	◯	◯

Biosicherheit

Was beinhaltet bei Ihnen Bestandsbetreuung auf diesem Gebiet bzw. in welchem Ausmaß gibt es Beratung?

*

Beantworten Sie diese Frage nur, wenn folgende Bedingungen erfüllt sind:
Antwort war 'Ja' *oder* 'Unsicher' bei Frage '102 [HBiosicherheit]' (Biosicherheit Werden Sie zu diesem Thema von Ihrem Tierarzt betreut? ())

Bitte wählen Sie die zutreffende Antwort für jeden Punkt aus:

	Ja	Unsicher	Nein
**Erstellung von HACCP Konzepten**	◯	◯	◯
**Beratung zu Impfstrategien**	◯	◯	◯
**Bereitstellen/ Mitbringen von betriebseigener Kleidung (Stiefel/ Overall)**	◯	◯	◯

Biosicherheit

Warum ist diese Rubrik nicht Teil Ihrer Bestandsbetreuung?

*

Beantworten Sie diese Frage nur, wenn folgende Bedingungen erfüllt sind:
Antwort war 'Nein' bei Frage '102 [HBiosicherheit]' (Biosicherheit Werden Sie zu diesem Thema von Ihrem Tierarzt betreut? ())

❶ Bitte wählen Sie eine der folgenden Antworten:
❶ Wenn Sie 'Sonstiges:' auswählen, spezifizieren Sie bitte Ihre Auswahl im entsprechenden Textfeld.
Bitte wählen Sie nur eine der folgenden Antworten aus:

- ◯ Ein anderer Berater führt dies durch.
- ◯ Das kostet mir zu viel Zeit.
- ◯ Ich habe daran kein Interesse/ keine Probleme.
- ◯ Ich bezweifle, dass der Tierarzt über ausreichend Wissen und Expertise verfügt
- ◯ Es ist zu teuer, wenn der Tierarzt das macht.
- ◯ Mein Tierarzt hat es noch nie angeboten bzw. ich wusste nicht, dass das möglich ist.
- ◯ Sonstiges

Biosicherheit

Was würden Sie sich wünschen bzw. was fehlt Ihnen für eine gute Bestandsbetreuung?

Beantworten Sie diese Frage nur, wenn folgende Bedingungen erfüllt sind:
Antwort war 'Ja' *oder* 'Unsicher' *oder* 'Nein' bei Frage '102 [HBiosicherheit]' (Biosicherheit Werden Sie zu diesem Thema von Ihrem Tierarzt betreut? ())

Bitte geben Sie Ihre Antwort hier ein:

Betriebswirtschaftlichkeit

Werden Sie zu diesem Thema von Ihrem Tierarzt betreut?

*

Bitte wählen Sie die zutreffende Antwort für jeden Punkt aus:

	Ja	Unsicher	Nein
	◯	◯	◯

Betriebswirtschaftlichkeit

Was beinhaltet bei Ihnen Bestandsbetreuung auf diesem Gebiet bzw. in welchem Ausmaß gibt es Beratung?

*

Beantworten Sie diese Frage nur, wenn folgende Bedingungen erfüllt sind:
Antwort war 'Ja' *oder* 'Unsicher' bei Frage '106 [HBetriebswirtschaft]' (Betriebswirtschaftlichkeit Werden Sie zu diesem Thema von Ihrem Tierarzt betreut? ())

Bitte wählen Sie die zutreffende Antwort für jeden Punkt aus:

	Ja	Unsicher	Nein
**Durchführung von Kosten-Nutzen-Analysen**	○	○	○
**Beratung zu Neuinvestitionen**	○	○	○
**stetige Optimierung/ Effizienz von Betriebsprozessen**	○	○	○

Betriebswirtschaftlichkeit

Warum ist diese Rubrik nicht Teil Ihrer Bestandsbetreuung?

*

Beantworten Sie diese Frage nur, wenn folgende Bedingungen erfüllt sind:
Antwort war 'Nein' bei Frage '106 [HBetriebswirtschaft]' (Betriebswirtschaftlichkeit Werden Sie zu diesem Thema von Ihrem Tierarzt betreut? ())

❗ Bitte wählen Sie eine der folgenden Antworten:
❗ Wenn Sie 'Sonstiges:' auswählen, spezifizieren Sie bitte Ihre Auswahl im entsprechenden Textfeld.
Bitte wählen Sie nur eine der folgenden Antworten aus:

- ○ Ein anderer Berater führt dies durch.
- ○ Das kostet mir zu viel Zeit.
- ○ Ich habe daran kein Interesse/ keine Probleme.
- ○ Ich bezweifle, dass der Tierarzt über ausreichend Wissen und Expertise verfügt
- ○ Es ist zu teuer, wenn der Tierarzt das macht.
- ○ Mein Tierarzt hat es noch nie angeboten bzw. ich wusste nicht, dass das möglich ist.
- ○ Sonstiges ______

Betriebswirtschaftlichkeit

Was würden Sie sich wünschen bzw. was fehlt Ihnen für eine gute Bestandsbetreuung?

Beantworten Sie diese Frage nur, wenn folgende Bedingungen erfüllt sind:
Antwort war 'Ja' *oder* 'Unsicher' *oder* 'Nein' bei Frage '106 [HBetriebswirtschaft]' (Betriebswirtschaftlichkeit Werden Sie zu diesem Thema von Ihrem Tierarzt betreut? ())

Bitte geben Sie Ihre Antwort hier ein:

______

Tierwohl

Werden Sie zu diesem Thema von Ihrem Tierarzt betreut?

*

Bitte wählen Sie die zutreffende Antwort für jeden Punkt aus:

	Ja	Unsicher	Nein
	○	○	○

Tierwohl

Was beinhaltet bei Ihnen Bestandsbetreuung auf diesem Gebiet bzw. in welchem Ausmaß gibt es Beratung?

*

Beantworten Sie diese Frage nur, wenn folgende Bedingungen erfüllt sind:
Antwort war 'Ja' *oder* 'Unsicher' bei Frage '110 [HTierwohl]' (Tierwohl Werden Sie zu diesem Thema von Ihrem Tierarzt betreut? ())

Bitte wählen Sie die zutreffende Antwort für jeden Punkt aus:

	Ja	Unsicher	Nein
**tierindividuelle Entscheidung bei Krankheit (z.B. klarer Rat zur Euthanasie)**	◯	◯	◯
**Beurteilung/ Beratung zum Verschmutzungsgrad der Tiere**	◯	◯	◯
**Beurteilung von sog. Technopathien (Liegestellen)**	◯	◯	◯
**Beurteilung des Liegeboxenkomforts**	◯	◯	◯
**Beurteilung der Liegeboxenhygiene**	◯	◯	◯
**Schulung zu tiergerechtem Umgang (z.B. Stockmanship)**	◯	◯	◯

Tierwohl

Warum ist diese Rubrik nicht Teil Ihrer Bestandsbetreuung?

*

Beantworten Sie diese Frage nur, wenn folgende Bedingungen erfüllt sind:
Antwort war 'Nein' bei Frage '110 [HTierwohl]' (Tierwohl Werden Sie zu diesem Thema von Ihrem Tierarzt betreut? ())

❶ Bitte wählen Sie eine der folgenden Antworten:
❶ Wenn Sie 'Sonstiges:' auswählen, spezifizieren Sie bitte Ihre Auswahl im entsprechenden Textfeld.
Bitte wählen Sie nur eine der folgenden Antworten aus:

- ◯ Ein anderer Berater führt dies durch.
- ◯ Das kostet mir zu viel Zeit.
- ◯ Ich habe daran kein Interesse/ keine Probleme.
- ◯ Ich bezweifle, dass der Tierarzt über ausreichend Wissen und Expertise verfügt
- ◯ Es ist zu teuer, wenn der Tierarzt das macht.
- ◯ Mein Tierarzt hat es noch nie angeboten bzw. ich wusste nicht, dass das möglich ist.
- ◯ Sonstiges

Tierwohl

Was würden Sie sich wünschen bzw. was fehlt Ihnen für eine gute Bestandsbetreuung?

Beantworten Sie diese Frage nur, wenn folgende Bedingungen erfüllt sind:
Antwort war 'Ja' *oder* 'Unsicher' *oder* 'Nein' bei Frage '110 [HTierwohl]' (Tierwohl Werden Sie zu diesem Thema von Ihrem Tierarzt betreut? ())

Bitte geben Sie Ihre Antwort hier ein:

Gibt es sonstige Bereiche, die noch im Rahmen der ITB besprochen werden? *

❶ Bitte wählen Sie eine der folgenden Antworten:
❶ Wenn Sie 'Ja, z.B. folgende:' auswählen, spezifizieren Sie bitte Ihre Auswahl im entsprechenden Textfeld.
Bitte wählen Sie nur eine der folgenden Antworten aus:

- ◯ Nein
- ◯ Ja, z.B. folgende:

z.B. Angestelltenmanagement, ...

Bitte suchen Sie aus den folgenden Bereichen 3 aus, die Sie persönlich am Wichtigsten finden.
Fangen Sie dazu mit dem für Sie wichtigsten Bereich (= 1) an und markieren in absteigender Reihenfolge.
Außerdem suchen Sie bitte den Bereich aus, den Sie selbst am Unwichtigsten als Aufgabe des ITB-Tierarztes empfinden.
Die übrigen Bereiche belassen Sie bitte auf "keine Antwort".
*

Bitte wählen Sie die zutreffende Antwort für jeden Punkt aus:

	1	2	3	11 (am Unwichtigsten)	keine Antwort
**Fruchtbarkeit**	◯	◯	◯	◯	◯
**Eutergesundheit**	◯	◯	◯	◯	◯
**Leistung/ Auswertung von Herdendaten/ Digitalisierung im Kuhstall**	◯	◯	◯	◯	◯
**Klauengesundheit**	◯	◯	◯	◯	◯
**Jungtiergesundheit**	◯	◯	◯	◯	◯
**Tierernährung (Fütterungs- und Leistungsanalysen)**	◯	◯	◯	◯	◯
**Stallbau/ Tierhaltung**	◯	◯	◯	◯	◯
**Biosicherheit**	◯	◯	◯	◯	◯
**Betriebswirtschaftlichkeit**	◯	◯	◯	◯	◯
**Tierwohl**	◯	◯	◯	◯	◯
**Angestelltenmanagement/ -ausbildung**	◯	◯	◯	◯	◯

In welchem Bundesland leben Sie? *

❗ Bitte wählen Sie eine der folgenden Antworten:
Bitte wählen Sie nur eine der folgenden Antworten aus:

- ◯ Baden-Würtemberg
- ◯ Bayern
- ◯ Berlin
- ◯ Brandenburg
- ◯ Bremen
- ◯ Hamburg
- ◯ Hessen
- ◯ Mecklenburg-Vorpommern
- ◯ Niedersachsen
- ◯ Nordrhein-Westfalen
- ◯ Rheinland-Pfalz
- ◯ Saarland
- ◯ Sachsen
- ◯ Sachsen-Anhalt
- ◯ Schleswig-Holstein
- ◯ Thüringen

Welches Geschlecht haben Sie? *

Bitte wählen Sie nur eine der folgenden Antworten aus:

- ◯ weiblich
- ◯ männlich

Welchem Altersintervall gehören Sie an? *

❶ Bitte wählen Sie eine der folgenden Antworten:
Bitte wählen Sie nur eine der folgenden Antworten aus:

- ◯ jünger als 30 Jahre
- ◯ 30 - 49 Jahre
- ◯ 50 - 65 Jahre
- ◯ älter als 65 Jahre

Welche Position üben Sie im Betrieb aus? *

❶ Bitte wählen Sie eine der folgenden Antworten:
Bitte wählen Sie nur eine der folgenden Antworten aus:

- ◯ Betriebsleiter
- ◯ Betriebsnachfolger
- ◯ Angestellte/r Herdenmanager/in
- ◯ Sonstiges ____________

Was ist Ihr derzeit höchster Abschluss? *

❶ Bitte wählen Sie eine der folgenden Antworten:
Bitte wählen Sie nur eine der folgenden Antworten aus:

- ◯ kein Schulabschluss
- ◯ Schulabschluss
- ◯ ausgebildete/r Herdenmanager/in
- ◯ Landwirtschaftliche Lehre: Geselle
- ◯ Landwirtschaftliche Lehre: Meister
- ◯ Fachhochschulstudium/ Höhere Landbauschule
- ◯ Universitätsstudium
- ◯ Sonstiges ____________

Was ist Ihr Zukunftsplan und wo sehen Sie sich in 10 Jahren? *

❶ Bitte wählen Sie eine der folgenden Antworten:
Bitte wählen Sie nur eine der folgenden Antworten aus:

- ◯ Betrieb weiterführen wie gehabt
- ◯ Anzahl melkender Kühe erweitern
- ◯ Anzahl melkender Kühe verringern
- ◯ Betrieb an einen Nachfolger übergeben
- ◯ Betrieb aufgeben
- ◯ Betrieb anderweitig umstrukturieren (z.B. Umstellung auf Biobetrieb/ ökologische Landwirtschaft, ...)
- ◯ Ich weiß es nicht.
- ◯ Sonstiges ____________

Würden Sie einen "Tag des offenen Stalls" durchführen? *

Bitte wählen Sie nur eine der folgenden Antworten aus:

- ◯ Ja
- ◯ Nein

Möchten Sie uns zum Schluss noch etwas mitteilen?

Bitte geben Sie Ihre Antwort hier ein:

____

Wenn Sie an der freiwilligen Verlosung der Fachzeitschriften-Abonnements teilnehmen möchten, können Sie uns hier Ihre Email-Adresse hinterlassen, über die wir Sie im Falle des Gewinns kontaktieren.

Die Verlosung erfolgt vollständig unabhängig von Ihren zuvor übermittelten anonymen Antworten.

Viel Glück!

Bitte geben Sie Ihre Antwort hier ein:

Vielen Dank für Ihre Teilnahme!

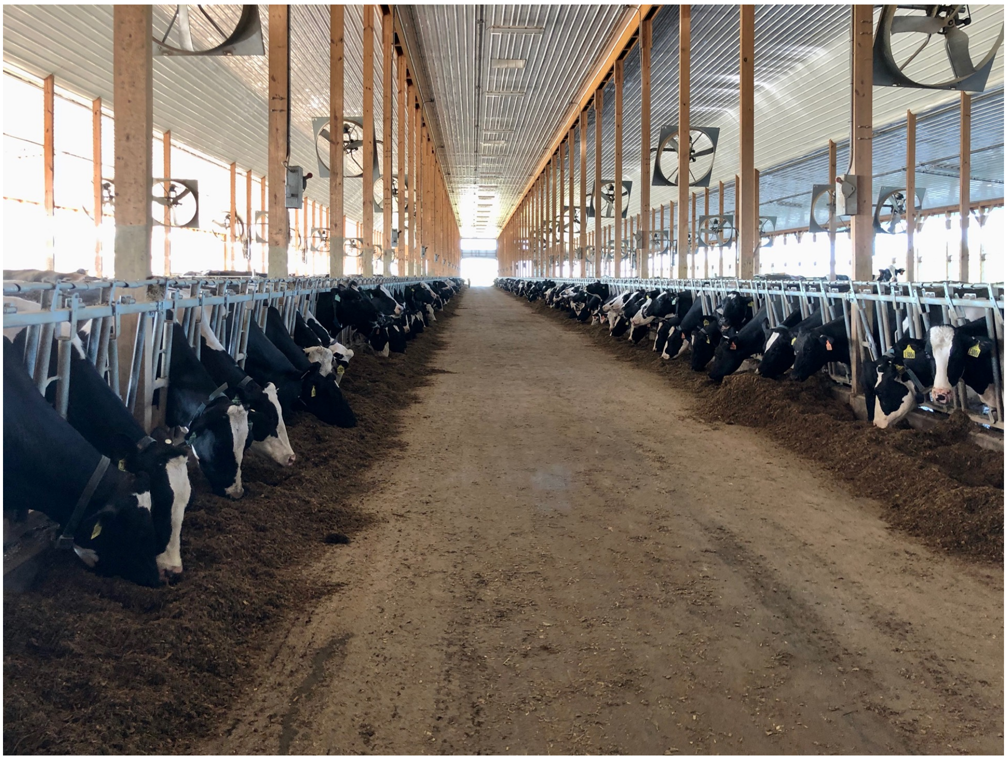

03.01.2021 – 10:15

Übermittlung Ihres ausgefüllten Fragebogens:
Vielen Dank für die Beantwortung des Fragebogens.
